# Reproductive biology of female common dolphins (*Delphinus delphis*) in New Zealand waters

**DOI:** 10.1007/s00227-022-04139-3

**Published:** 2022-11-28

**Authors:** Emily I. Palmer, Emma L. Betty, Sinéad Murphy, Matthew R. Perrott, Adam N. H. Smith, Karen A. Stockin

**Affiliations:** 1 Cetacean Ecology Research Group, School of Natural Sciences, Massey University, Auckland, 0745 New Zealand; 2 Department of Natural Resources and the Environment, Marine and Freshwater Research Centre, School of Science and Computing, Atlantic Technological University, ATU Galway City, Old Dublin Road, Galway, H91 T8NW Ireland; 3 School of Veterinary Science, Massey University, Palmerston North, 4472 New Zealand; 4 School of Mathematical and Computational Sciences, Massey University, Auckland, 0745 New Zealand

**Keywords:** Life history, Reproduction, Density dependence factors, Fisheries interactions, Management, SDG14

## Abstract

Reproductive biology was assessed in 106 female common dolphins (*Delphinus delphis*) examined post-mortem from stranding and bycatch events along the New Zealand coastline between 1997 and 2019. The average age (ASM) and length (LSM) at sexual maturity was estimated at 7.5 years and 183.5 cm, respectively. The total number of *corpora* in mature individuals increased with age and appeared to persist throughout life. Ovarian asymmetry was apparent, with the left ovary displaying higher rates of ovulation, and a maximum of 19 *corpora* recorded for a 24-year-old female. The estimated ovulation and annual pregnancy rates for mature females were 0.39 year^−1^ and 30%, respectively. Conception and calving occurred year-round, with a weak seasonal increase observed in late austral spring and early austral summer. As these data did not clearly show whether seasonality was present, the gestation, lactation, and resting periods were calculated as either 12.6 or 12.8 months based on the presence/absence of seasonality, respectively. Similarly, calving interval ranged from 3.15 to 3.2 years, depending upon whether seasonality was considered. The estimated LSM of the New Zealand population aligns with other populations globally, although the estimated ASM is younger by approximately 6 months. Other reproductive parameters align with Northern Hemisphere populations, although demonstrate variation, which may reflect adaptations to local conditions such as water temperature and prey availability. As the species is subject to anthropogenic impacts including pollution and bycatch, we suggest our findings be used as a baseline with which to monitor trends in population parameters.

**Supplementary Information:**

The online version contains supplementary material available at 10.1007/s00227-022-04139-3.

## Introduction

Robust estimates of reproductive parameters, such as attainment of sexual maturity, gestation and lactation periods, annual pregnancy rates, and calving intervals, are essential for effective conservation and management (Lanyon and Burgess 2014; Rossi et al. 2017), especially for declining populations (Botta et al. 2010). Differences in reproductive parameters, mating strategies, and behaviour exist among cetacean species, with body size reported to play a role in this variation (Fedak et al. 2002; González‐Suárez and Revilla 2013). Females of small odontocete species, such as harbour porpoises (*Phocoena phocoena*), attain sexual maturity around 2–4 years (Learmonth et al. 2014; Kesselring et al. 2017; Murphy et al. 2020), whereas females of larger species, such as long-finned pilot whales (*Globicephala melas edwardii*) obtain sexual maturity at around 6–7 years of age (Betty 2019). In comparison, females of the largest odontocete species, sperm whales (*Physeter macrocephalus*), reach sexual maturity at approximately 9 years of age (Best et al. 1984).

Average age at attainment of sexual maturity (ASM) can vary across populations of the same species and within a population over time. Variation in ASM can reflect differences in reproductive potential and success and can, therefore, be used to identify populations at risk (Wade 2018). Annual pregnancy rate (APR) is another critical parameter when assessing the viability of populations since it indicates the proportion of sexually mature females in the population likely to be pregnant at any given time. Like ASM, APR can vary markedly among species. Striped dolphins (*Stenella coeruleoalba*) off the south-east coast of southern Africa have an estimated APR of 26% (Bishop 2014). In comparison, harbour porpoises across the Northern Hemisphere have APRs that range from 47–50 (Murphy et al. 2015) to 98% (Ólafsdóttir et al. 2003) depending on location and population. Variation in reproductive parameters within species may be driven by several factors including anthropogenic pressures and differences in habitat and availability of resources. For example, the types and amounts of prey available influence survival rates and, therefore, may influence the number of reproducing females, and the viability of their offspring, in a population (Wade 2018). In the case of the Indo-Pacific bottlenose dolphin (*Tursiops aduncus*), mortality rates of calves are higher in Adelaide, South Australia compared to other areas due to the intensity of anthropogenic impacts such as entanglement, pollution and boat strike (Steiner and Bossley 2008).

The reproductive biology of female common dolphins (*Delphinus delphis*) has been studied in a small number of populations in the Northern Hemisphere, especially in the North Atlantic (Westgate and Read 2007; Murphy et al. 2009) and eastern tropical Pacific (Danil and Chivers 2007). In contrast, there has been little examination of reproductive parameters in Southern Hemisphere populations, except for Argentina (Grandi et al. 2022). Notably, reproductive parameters remain unknown for female common dolphins in Oceania, despite documented anthropogenic impacts including bycatch in commercial fisheries in this region (Du Fresne et al. 2007; Ministry for Primary Industries 2016; Abraham and Berkenbusch 2017; Goldsworth et al. 2019; Barceló et al. 2021). Recent genomic insights suggest New Zealand and Australian common dolphin populations are substantially connected and should be considered a single fisheries management unit (Barceló et al. 2021). Monitoring trends in life history parameters of the management unit also allows for the monitoring of anthropogenic impacts. As there are no abundance estimates available for the whole management unit (Stockin and Orams 2009) and (annual) data on the total number of individuals bycaught within the unit is lacking, this is particularly important.

A common method of quantifying population viability is to calculate the maximum rate of increase (*r*_max_; Dans et al. 2003; Mannocci et al. 2012). If the incidental mortality rate exceeds the rate of increase (*r*_max_), then the population will decline (Dans et al. 2003). If abundance data are unavailable, then knowledge of key reproductive parameters are required to reliably estimate *r*_max_. Our understanding of the viability of both the New Zealand and Australian populations of common dolphins is currently hindered by a lack of knowledge, which is concerning given the number of observed fisheries bycatch events in the region (Thompson et al. 2013; Fisheries New Zealand 2020; Tulloch et al. 2020; Barceló et al. 2021; Parra et al. 2021).

Here, we estimate reproductive parameters for female common dolphins based on post-mortem data taken from stranded and bycaught female common dolphins in New Zealand waters. Specifically, we address (1) classification of reproductive status, (2) average age (ASM) and body length (LSM) at attainment of sexual maturity, (3) ovarian characteristics and persistence of scars, (4) reproductive phases, (5) annual pregnancy rate and calving interval, and (6) reproductive seasonality.

## Materials and methods

### Sample collection

**Fig. 1:**
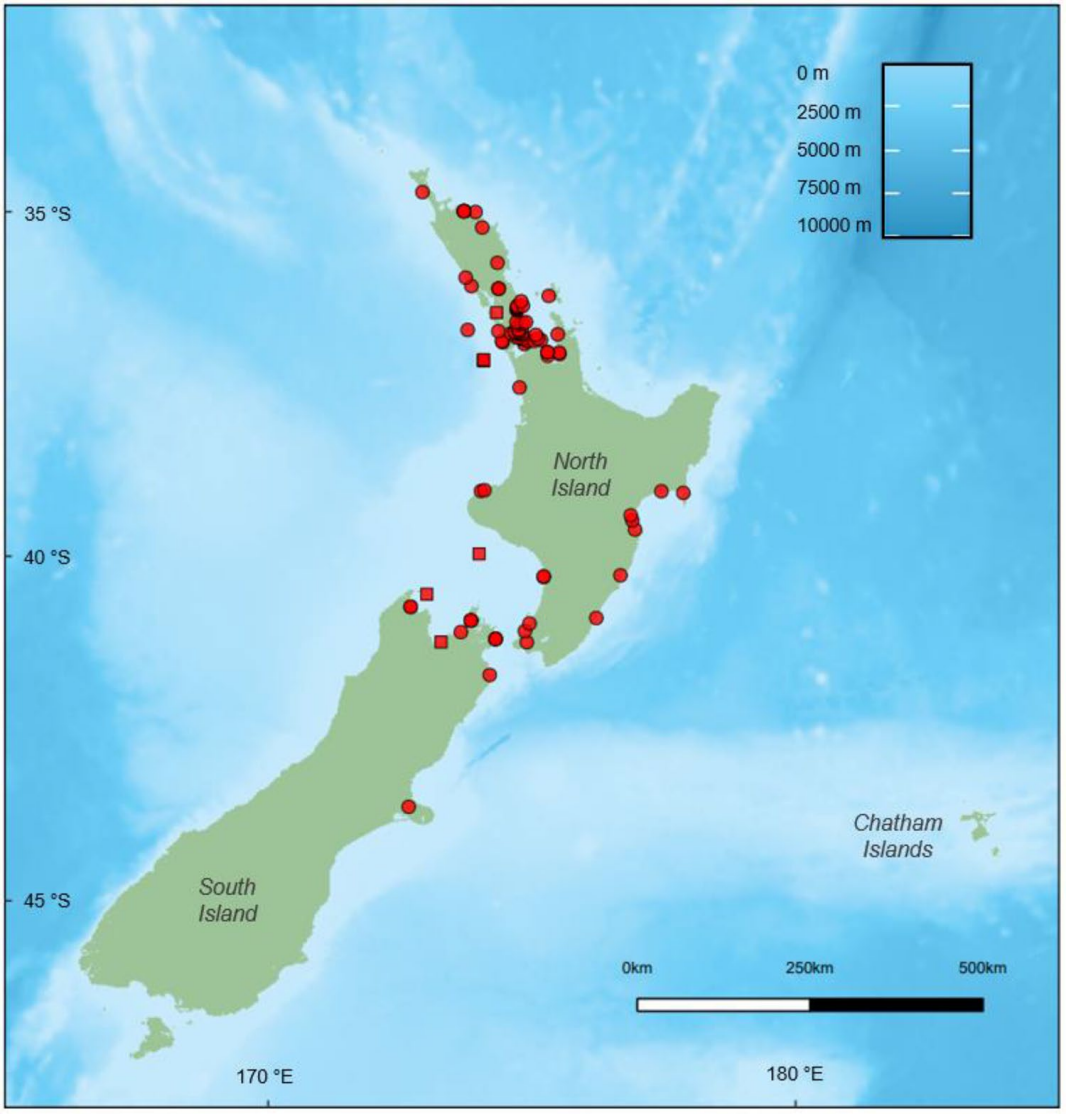
Location of common dolphin (*Delphinus delphis*) stranding (red circles) and bycatch (red squares) events around New Zealand, from which female reproductive samples were collected for this study (*n* = 101)

Reproductive data were collected post-mortem from a total of 106 female common dolphins following Geraci and Lounsbury (2005). The sample included 97 dolphins that had either live-stranded or were found beachcast during 75 independent events along the New Zealand coastline between 1997 and 2019. A further 9 bycaught dolphins from 7 incidental captures in the commercial jack mackerel (*Trachurus novaezelandiae*) fishery off the west coast of North Island of New Zealand between 1999 and 2003 (Fig. 1) were obtained. As this is an opportunistic dataset with a limited sample size, there is a possibility that the sample may not be fully representative of the population.

Ovaries and the associated reproductive tract were removed and examined grossly. Ovaries were initially examined for the presence of *corpora albicantia* (CAs) and *corpora lutea* (CLs), with photographs taken in situ. The ovaries were subsequently extracted from the reproductive tract and additional photographs were taken, with emphasis on scars or prominent ovarian features. Ovaries were weighed (g) and measured (mm) before being fixed in 10% neutral buffered formalin. A foetus in the uterus indicated a pregnancy. The foetus was then measured (crown to rump) to the nearest mm, weighed to the nearest g, photographed, and where possible, sexed anatomically. The mammary glands were examined for evidence of lactation by pressing externally around the mammary slit and noting any fluid emission, and internally by cross-sectioning the gland and noting the presence of milk. Not all variables were measured from each carcass and so sample sizes vary.

Teeth were extracted and collected for age determination following methods summarised in Murphy et al. (2014). The straightest, least worn teeth were selected from the middle of the bottom jaw. Total body length (TBL) and associated morphometrics were measured to the nearest 0.5 cm. Decomposition state was noted for each individual (*fresh*, *mild*, and *moderate*) as per Stockin et al. (2007).

### Age estimation

Age was estimated by examining decalcified, stained thin sections of teeth from each individual (Murphy et al. 2014). Sections were examined using a binocular microscope (10–40 × magnification), and the age was estimated by counting the annual growth layer groups (GLGs) in the dentine as described by Lockyer (1995) and Myrick et al. (1983). Tooth sections were initially read blind (i.e., with no prior biological information known), by at least two of three experienced readers (SM, EB, EP). Best age estimates or age ranges were subsequently compared and in the case of any disagreement, further teeth were prepared and examined until a final estimate was determined (Murphy et al. 2014). Individuals that could not be reliably aged (i.e., due to tooth wear or damage) were excluded from further analysis. A neonate was identified if the neonatal line was not present or just forming in the dentine of the tooth.

### Reproductive status

Female reproductive status was determined through the assessment of ovaries, uteri and mammary glands, as outlined in Murphy et al. (2009). Females were considered sexually mature if there was at least one CA or CL present on the ovaries and/or they were pregnant and/or lactating, otherwise they were considered sexually immature. For mature females, reproductive status was classified according to Perrin and Donovan (1984) as follows: (1) pregnant, when a foetus is present in the uterus and a CL is present on one ovary, (2) pregnant and lactating, where a foetus is observed, a CL is present on one ovary, and milk is being produced (detected in the mammary glands), (3) lactating, milk is being produced, and (4) resting mature, a sexually mature female that is neither pregnant nor lactating.

### Average age and body length at attainment of sexual maturity

The ASM and LSM were estimated for female common dolphins using two methods: (1) Bayesian modelling (Huisman et al. 1993) and (2) the sum-of-fraction of immature (SOFI) method (Hohn 1989, see S8 in Supplementary Material).

Female ASM and LSM were modelled using Bayesian logistic regression with ‘HOF’ parameterisation (Huisman et al. 1993), fitted with Stan (Stan Development Team 2021) in R (R Development Core Team 2021). The HOF equation is as follows:

$$P\left( {y = 1|x, m,\omega } \right) = \frac{1}{{1 + \exp \left( { - \omega \left( {x - m} \right)} \right)}},$$

where *y* is maturity status (0 = immature; 1 = mature), and *x* is either age or TBL. The two model parameters were a slope *ω* and a midpoint *m*, representing the age or length at which the probability of sexual maturity is 0.5 (i.e., the median age or length at attainment of sexual maturity). Prior distributions for the model parameters were *m* ~ Normal (190,20), *m* ~ Normal^+^ (0,1) for TBL, and *m* ~ Normal (8,2), *ω* ~ Normal^+^ (0,2) for age. Prior predictive modelling was done to ensure these priors were reasonable.

The model was fitted to age and length data separately to obtain the ASM and LSM. The utility of age and TBL (*n* = 83 complete cases) for predicting maturity status were compared using the Leave-One-Out Expected Log Predictive Density (ELPD) and estimated using Pareto-smoothed importance sampling (Vehtari et al. 2017). ELPD scores give a measure of predictive accuracy for out-of-sample data.

### Ovarian assessment

Prior to examination, formalin-fixed ovaries were rinsed in water for 24 h and transferred back to containers with 70% ethanol. For each ovary, maximum length, width, and depth were measured and recorded to the nearest 0.1 mm using vernier callipers. The weight of each ovary was recorded to the nearest 0.1 g. Combined ovarian weight was calculated for females where both ovaries were collected (*n* = 92). If a CL was present (> 9 mm in diameter and yellow pigmentation), the position of the CL was recorded. Each ovary was then hand sectioned into 2 mm slices and examined under a 5 × magnifying lamp to count the total number of *corpora* present. Ovaries were sectioned with the hilar region (where the ovaries attach to a broad ligament of the uterus; Saksouk and Johnson 2004), left intact to hold the sections together. The diameters of any CLs and CAs, and the largest follicle were measured to the nearest 0.1 mm on three planes perpendicular to each other using vernier callipers.

The activity of both ovaries via *corpora* was recorded to assess symmetry. *Corpora* data were tested for normality with a Shapiro–Wilk test. As these data were not normally distributed, a Kruskal–Wallis test was applied to compare the total *corpora* count between left and right ovaries, testing the null hypothesis of ovarian symmetry. A linear regression was additionally applied to assess if CL size increased with foetus TBL.

### Ovulation rate

The total count of *corpora* (CL + CAs) scars was used to provide an indication of ovulation rate, as *corpora* count typically increases with size and/or age for mature females (Boyd et al. 1999; Takahashi et al. 2006; Ellis et al. 2018). Age and TBL were regressed against *corpora* count to determine the persistence of ovulation scars. The ovulation rate was subsequently estimated by regressing mean *corpora* count on age. This is under the assumption that CAs persist indefinitely (Danil and Chivers 2007; Westgate and Read 2007; Murphy et al. 2010; Betty 2019) and the slope of the regression corresponds to the rate at which the *corpora* are formed (Perrin and Reilly 1984).

### Length at birth

Median length at birth was modelled using the Bayesian logistic regression with ‘HOF’ parameterisation (Huisman et al. 1993) described above. After prior predictive simulations, the following weakly informative priors were chosen for the two parameters, *m* ~ N (90,20), *ω* ~ N^+^ (0,1). The dataset of this model included all foetuses and postnatal dolphins ≤ 160 cm (*n* = 103) for which TBL was available. This dataset comprised of 13 unborn (*n*_0_) and 90 born dolphins (*n*_1_). To mitigate any potential effects of the unbalanced sample on estimates (Salas-Eljatib et al. 2018), weights were assigned to each case. These cases were from group *k* according to the sample size of the group *n*_*k*_, relative to the overall sample size, *n*, using $w_{k} = \sqrt {0.5/\left( \frac{nk}{n} \right)}$. For the weights *w*_*i*_ to have an average of 1, the vector of weights *w*_*i*_ were normalised.

Two models were fit, the fully weighted model and the square-root weighted model. These models were compared using model weights and Leave-One-Out diagnostics (via Pareto-smoothed importance sampling and refitting models for 103 observations with Pareto *k* > 0.7; ‘loo’ package in *R*; Vehtari et al. 2017). The partially weighted model, taking the square root of the sample size difference, was a better fit and so was used. The posterior distribution of the parameter *m* (i.e., the length at which the probability of birth is 50%) was summarised by the mean and 95% highest posterior-density credible interval.

### Gestation period and foetal growth

A plot of total body length (TBL) of foetuses and neonatal calves against day of year of collection (Julian date) was created for the New Zealand dataset (Perrin and Reilly 1984, see S1 in Supplementary Material). The plot indicated that births were not clearly synchronised and/or gestation length was longer than 12 months as both very small and very large foetuses were recorded between April and July (Julian dates 103–202). Following Martin and Rothery (1993), these data were copied three times to mimic three consecutive ‘years’ to assess the temporal spread of conceptions. Three orientated concentrations of points from smallest to largest foetuses were clearly identified, which provided a basis for allocating a ‘cohort’ to foetal specimens and suggested there may be a seasonal component to conceptions and births (Figs. 7, S1).

Gestation period was estimated two ways: (1) Huggett and Widdas (1951) nonlinear growth phase method, adapted by Laws (1959), and (2) Perrin et al. (1977) gestation regression equation. The dataset fits the assumptions required for the two different equations (presence vs. absence of seasonality), hence why both were used to calculate the gestation period for New Zealand common dolphins.

The Huggett and Widdas (1951) equation assumes that seasonality is present in the dataset and is calculated as follows:

$${\text{Total gestation period}}\,\left( {t_{{\text{g}}} } \right) = t_{0} + \left( {t_{{\text{g}}} - t_{0} } \right),$$

where *t*_0_ is the nonlinear phase of growth, and (*t*_g_ − *t*_0_) is the linear phase of growth.

Using the Huggett and Widdas (1951) equation, the linear phase of growth (*t*_g_ − *t*_0_) was calculated, using 87.6 cm (95% CrI: 81.3–92.6 cm) as the best estimate of length at birth. An estimate of nonlinear growth for this study is reliant on previously published estimates of the relationship of *t*_0_ to *t*_g_, or (*t*_g_ − *t*_0_). This is because it cannot be ruled out that some exceptionally small embryos were missed from the data collection. Furthermore, previous calculations of nonlinear growth in common dolphins also applied mass as a parameter (Westgate and Read 2007; Murphy et al. 2009). While mass was not systematically collected in our study, the proportion of nonlinear to linear growth phase (0.126) reported in previous studies was used to calculate the nonlinear growth phase for New Zealand common dolphins.

The Perrin et al. (1977) regression equation was also used as there was evidence from our dataset that conceptions and births may not have a seasonal component. This method is used when there is no reproductive seasonality observed and is as follows:

$${\text{Log}}\left( y \right) = 0.1659 + 0.4856 \, \log (x),$$

where (*y*) is the length of gestation in months and (*x*) is the length at birth in cm.

### Lactation period, length at weaning, and resting period

The lactation period was calculated as the proportion of lactating females divided by the proportion of pregnant females in the sample, multiplied by the gestation period expressed in years:

$${\text{Lactation period}}\,\left( {t_{l} } \right) = t_{{\text{g}}} \times l/p,$$

where *t*_g_ is the length of gestation, *l* is the proportion of the sample lactating, *p* is the proportion of the sample pregnant (including females simultaneously pregnant and lactating; Perrin and Reilly 1984).

The length at weaning was calculated using the Huang et al. (2009) equation:

$${\text{Length at weaning}}\,\left( {L_{{\text{w}}} } \right) = 1.2399L_{x}^{0.877} ,$$

where *L*_*x*_ is the female asymptotic length (cm).

The resting period was calculated as the proportion of resting females divided by the proportion of pregnant females in the sample, multiplied by the gestation period expressed in years:

$${\text{Resting period}}\,\left( {t_{{\text{r}}} } \right) = t_{{\text{g}}} \times r/p,$$

where *t*_r_ is the length of the resting period, *t*_g_ is the length of gestation, *r* is the proportion of the sample resting, and *p* is the proportion of the sample pregnant (including females simultaneously pregnant and lactating; Perrin and Reilly 1984).

#### Annual pregnancy rate and calving interval

The annual pregnancy rate (APR) was estimated by dividing the proportion of pregnant females in the sample by the length of gestation, expressed in years (Murphy et al. 2009; Perrin and Reilly 1984):

$${\text{Annual pregnancy rate}}\,\left( {{\text{APR}}} \right) = p/t_{{\text{g}}} ,$$

where *t*_g_ is the length of gestation, and *p* is the proportion of sample pregnant (including females that were simultaneously pregnant and lactating).

Several assumptions are implicit in this model: (1) there is no sampling bias, i.e., the reproductive condition in the sample is the same as the population that is being sampled, (2) there is no seasonal bias that exists within the sample collection, (3) all pregnancies are detected (Perrin and Reilly 1984), and (4) that the length of gestation is calculated accurately. To avoid missing the presence of early embryos, it is recommended to exclude samples collected during the mating period (Murphy et al. 2009). However, as no clear mating season was established for the population and given the sample size, all data were included. To follow previous recommendations, an additional calculation of gestation period was performed with the exclusion of individuals sampled between August and November (slight calving peak).

The calving interval (CI) was calculated in two ways: (1) the reciprocal of the APR and (2) the summation method (gestation + lactation + resting phases) after Perrin et al. (1977). For summation, the length of lactation needs to be adjusted downward by a factor equal to the percentage of females that are simultaneously pregnant and lactating. This considers any overlap between pregnancy and lactation (Perrin et al. 1977).

#### Reproductive seasonality

Conception and birth dates were estimated for each foetus and yearling calf (i.e., < 1 year old), based on their estimated age (Börjesson and Read 2003).

$$\begin{aligned} & {\text{Foetal}}\,\left( t \right) = \left( {L_{{\text{t}}} /u} \right) \times 30.5 + t_{0} \\ & {\text{Yearling calf}}\,\left( t \right) = \left( {L_{{\text{t}}} - L_{{\text{b}}} /\left( {u/30.5} \right)} \right), \\ \end{aligned}$$

where *t* is the foetal/calf age in days, *L*_t_ is the actual length of the foetus/calf (cm), *u* is the appropriate foetal, male calf, or female calf growth rate (cm mo^−1^), 30.5 is the average number of days in a month, *t*_0_ is the nonlinear foetal growth rate, and *L*_b_ is the estimated length-at-birth.

Calf age was estimated using 87.6 cm as the average length-at-birth, and the length at one year of age was 133.6 cm (95% CI 128.7–138.5 cm) for females and 139.8 cm (95% CI 132.5–147.1 cm) for males (determined from sex-specific growth curves; Palmer et al. unpublished data). From this, the first-year growth rate for female and male common dolphins was estimated at 46 cm/year and 52.2 cm/year, respectively. The nonlinear foetal growth rate was calculated based on the proportion of nonlinear/linear growth phases as in Murphy et al. (2009). Using the Huggett and Widdas method for calculating gestation, the nonlinear growth phase was 43 days and *u* was 6.95 cm/month. Using the regression method, the nonlinear growth phase was 44 days and *u* was 6.84 cm/month. Yearling calves (*n* = 32) were chosen if they were aged < 1 year or below, by counting GLGs in the dentine of the teeth (Palmer et al. unpublished data).

Individual conception dates for foetuses were calculated by subtracting the estimated foetal age (*t* in days) from the date stranded (Julian date). Birth dates for foetuses were estimated by adding the estimated conception date to the estimated gestation length (Huggett and Widdas method = 384 days or 12.6 months; regression method = 391 days or 12.8 months). Birth dates for calves were calculated by subtracting the estimated calf age (*t* in days) from the date stranded (Julian date), and conception dates were estimated by subtracting the estimated gestation period from the estimated birth date.

Data such as stranding dates of neonatal calves and near-term foetuses, the presence of large follicles, and the number of ovulating females in the sample were also used in the assessment of reproductive seasonality.

## Results

### The sample

**Fig. 2:**
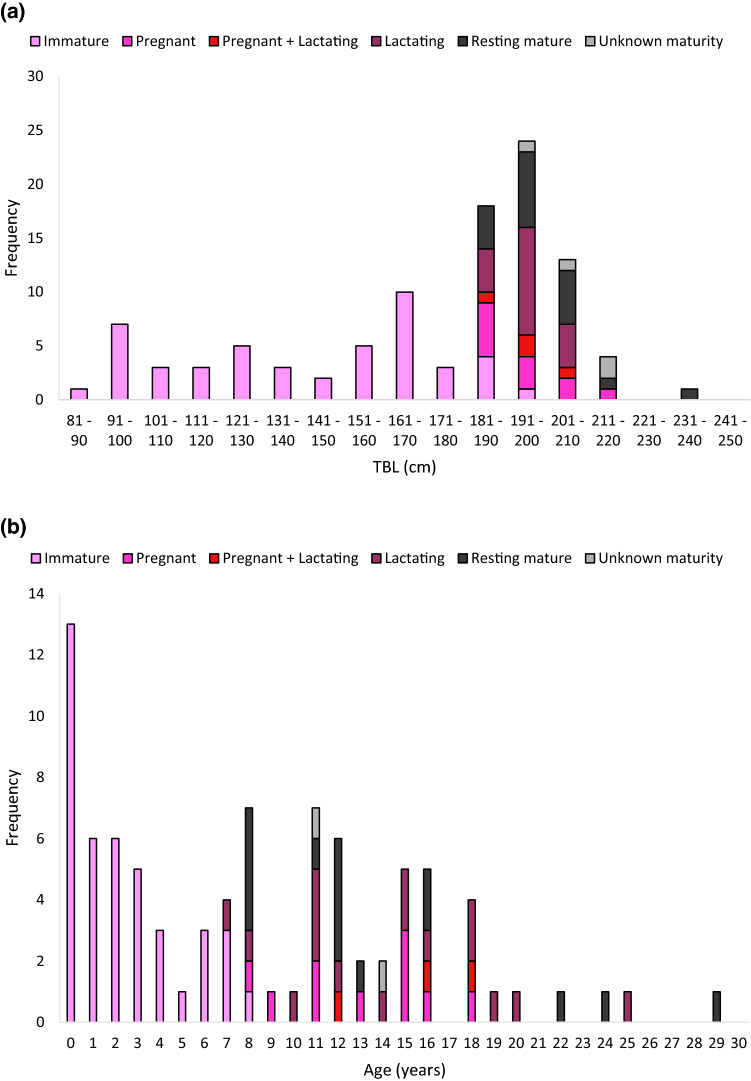
Frequency distribution of each maturity stage at **a** TBL (*n* = 104) and **b** age (*n* = 86) for female common dolphins (*Delphinus delphis*) stranded and bycaught on the New Zealand coast between 1997 and 2019

Females ranged from 82 to 233 cm in TBL (*x̄* = 177; SD = 33; *n* = 106), with a modal size class of 191–200 cm (Fig. 2a, Table 1). Age was determined for 100 female common dolphins, which ranged from 0 to 29 years (Fig. 2b, Table 1). The age distribution had a modal class of yearling calves (i.e., < 1 year old) and 83% of females (*n* = 88) were aged at 15 years or below.

**Table 1:** Mean (± SE), range, and number of samples obtained for total body length (TBL), age and ovarian characteristics of all reproductive groups of female common dolphins (*Delphinus delphis*) examined from New Zealand waters (1997–2019)

Stage	*n*	TBL (cm)	Age (years)	Total ovarian weight (g)	Total ovarian volume (mm^3^)	Corpora count (CAs + CL)		CL present	
						*L*	*R*	*L*	*R*
Immature	50	141 (± 5)	1.8 (± 0.3)	2.5 (± 0.5)	2.4 (± 0.8)	0	0	0	0
		82–193	0– ≥ 9	0.3–11.1	0.1–32.0				
		(*n* = 50)		(*n* = 40)	(*n* = 41)				
Pregnant	13	198 (± 3)	12.9 (± 1.0)	13.3 ± 1.0	11.2 ± 2.1	3.8 ± 1.2	2.6 ± 1.0	5	5
		185–216	8–18	7.8–17.1	4.6–20.9	0–12	0–9		
		(*n* = 13)	(*n* = 11)	(*n* = 10)	(*n* = 9)	(*n* = 10)	(*n* = 11)		
Lactating	18	196 (± 2)	14.4 (± 1.2)	6.2 (± 0.6)	5.5 (± 0.7)	4.6 (± 1.1)	0.7 (± 0.4)	5	2
		189–208	7–25	2.5–12.5	0.8–12.2	0–16	0–6		
		(*n* = 18)	(*n* = 17)	(*n* = 16)	(*n* = 17)	(*n* = 17)	(*n* = 17)		
Pregnant + lactating	4	197 (± 4)	15 (± 3.0)	13.9 (± 2.5)	8.2 (± 1.4)	5.5 (± 1.4)	1.5 (± 1.0)	2	2
		189–207	12–18	9.4–19	6.1–10.6	2–9	0–4		
		(*n* = 4)	(*n* = 3)	(*n* = 4)	(*n* = 4)	(*n* = 4)	(*n* = 4)		
Resting mature	18	199 (± 3)	14.2 (± 1.6)	6.5 (± 0.7)	9.7 (± 4.1)	5.9 (± 1.2)	2 (± 1.0)	3	2
		184–233	8—29	2.9–10.6	0.8–70.2	0–14	0–14		
		(*n* = 18)	(*n* = 16)	(*n* = 16)	(*n* = 16)	(*n* = 16)	(*n* = 16)		
Indeterminate mature	3	206 (± 6)	14 (± 0.0)	12.7 (± 9.4)	7.3 (± 1.9)	2.5 (± 2.5)	2.5 (± 2.5)	1	1
		200–212	14– > 20	3.3–22	5.4–9.2	0–5	0–5		
		(*n* = 3)	(*n* = 3)	(*n* = 2)	(*n* = 2)	(*n* = 2)	(*n* = 2)		
All mature individuals	56	198 (± 1)	14.4 (± 0.7)	8.7 (± 0.7)	8.3 (± 1.5)	4.8 (± 0.6)	1.7 (± 0.4)	16	12
		183–233	7–29	2.5–22	0.8–70.2	0–16	0–14		
		(*n* = 56)	(*n* = 51)	(*n* = 48)	(*n* = 48)	(*n* = 49)	(*n* = 50)		
Total	106	171 (± 4)	8.5 (± 0.7)	6.2 (± 0.6)	5.6 (± 0.9)	2.4 (± 0.4)	0.9 (± 0.2)	16	12
		82–233	0–29	0.3–22	0.1–70.2	0–16	0–14		
		(*n* = 106)	(*n* = 94)	(*n* = 88)	(*n* = 89)	(*n* = 96)	(*n* = 97)		

### Reproductive status

Reproductive status was determined for 106 females of which 47% (*n* = 50) were sexually immature and 53% (*n* = 56) were sexually mature (Table 1). Sexually immature individuals ranged from 82 to 193 cm in TBL and from 0 to 8.75 years in age. An additional immature female had a minimum age determined (> = 9 years) and was not included in the ASM calculations/modelling. Sexually mature females ranged from 183 to 233 cm in TBL and from 7 to 29 years in age. Of the mature sample (*n* = 56), 13 were pregnant, 18 were lactating, four were simultaneously pregnant and lactating, 18 were resting mature, and reproductive status was not determined for three mature females (Table 1). Of the four females that were simultaneously lactating and pregnant, three foetus ‘crown to rump’ measurements were recorded; 101 cm, 6.1 cm and 3.9 cm. This would suggest that one female was lactating for impending birth and two were assumed to be lactating from a previous pregnancy.

### Average age and length at attainment of sexual maturity

**Fig. 3:**
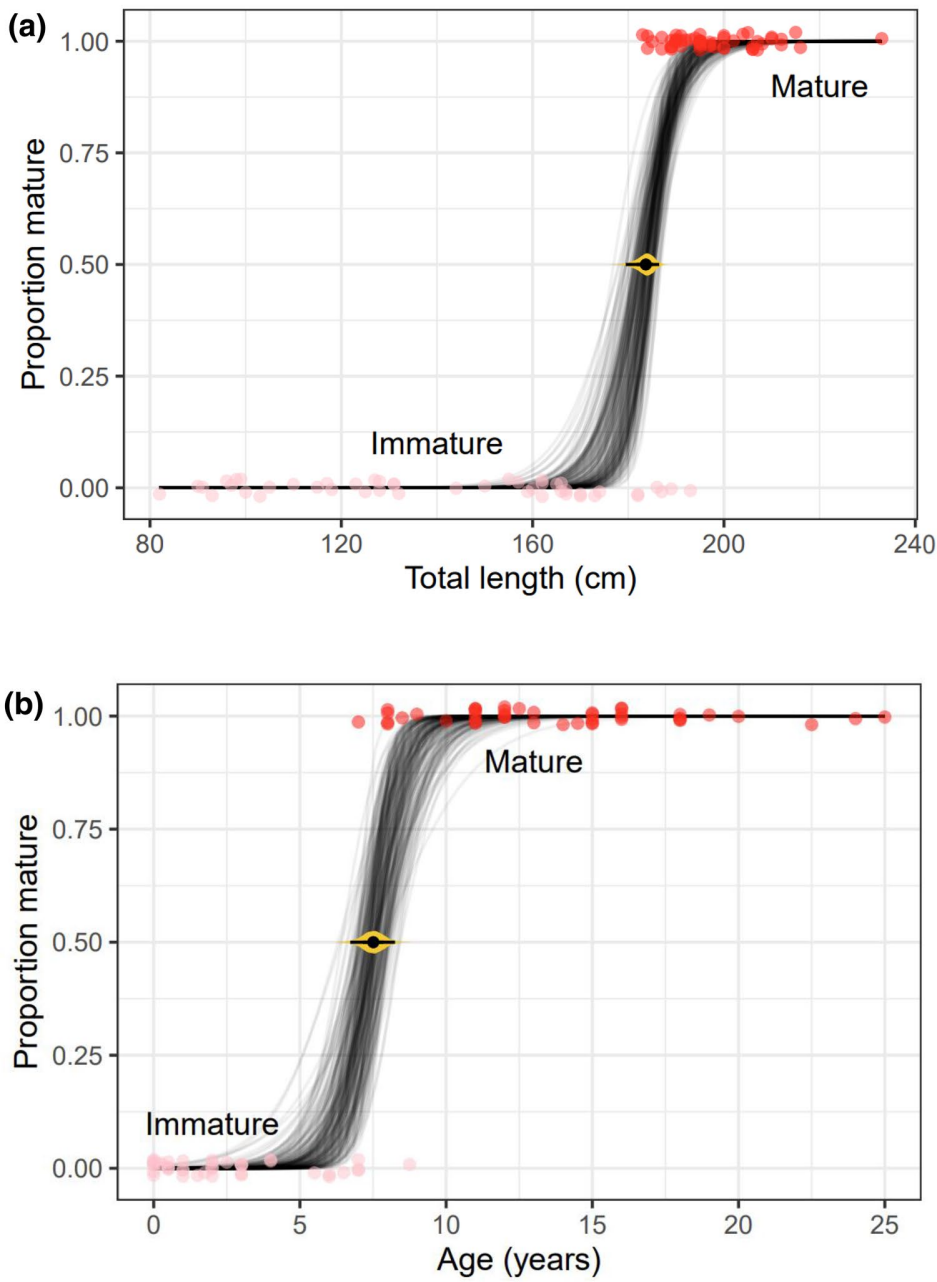
Bayesian cumulative logit regression of the sexual maturation of female common dolphins (*Delphinus delphis*) from New Zealand waters (1997–2019), modelled as a function of **a** total body length (TBL) and **b** age. These plots show the age and TBL values for immature (light pink) and mature (red) individuals. The lines represent posterior predictions of the mean. To aid the visualisation of overlapping points, a small amount of transparency and vertical ‘jitter’ was added. The central black point with the thin horizontal line shows the mean and 95% confidence intervals, with gradient plot in yellow (Kay 2021) of the estimated values of *x* at which 50% of females were classified as mature

Overlap was noted between immature and mature females of 183–193 cm TBL and 7–9 years of age (Table 1). Using the Bayesian logistic regression method, the LSM was estimated to be 183.5 cm [95% Credible Interval (CrI) = 179.5–186.5 cm, *n* = 100, Fig. 3a] and the ASM was estimated as 7.5 years (95% CrI = 6.7–8.3 yrs, *n* = 84, Fig. 3b). Using the SOFI method, the LSM and ASM were 188.9 cm (95% CI 187.9–189.9, *n* = 27) and 8.39 years (95% CI 7.25–9.53, *n* = 17, see S7 in Supplementary Material), respectively. The smallest sexually mature female (WS03-43Dd) measured 183 cm in TBL and had a maturity status of resting mature with a total of 12 *corpora* scars. The youngest sexually mature female (KS19-23Dd) was estimated to be 7 years old, was lactating, and the uterus exhibited signs of a recent pregnancy. A total of 12 *corpora* scars were recorded for this female. The ELPD score for the model with age (0, SE = 0) was significantly greater than the model with TBL (− 47.8, SE = 4), indicating that age was a better predictor of sexual maturity than TBL.

### Ovarian characteristics

**Fig. 4:**
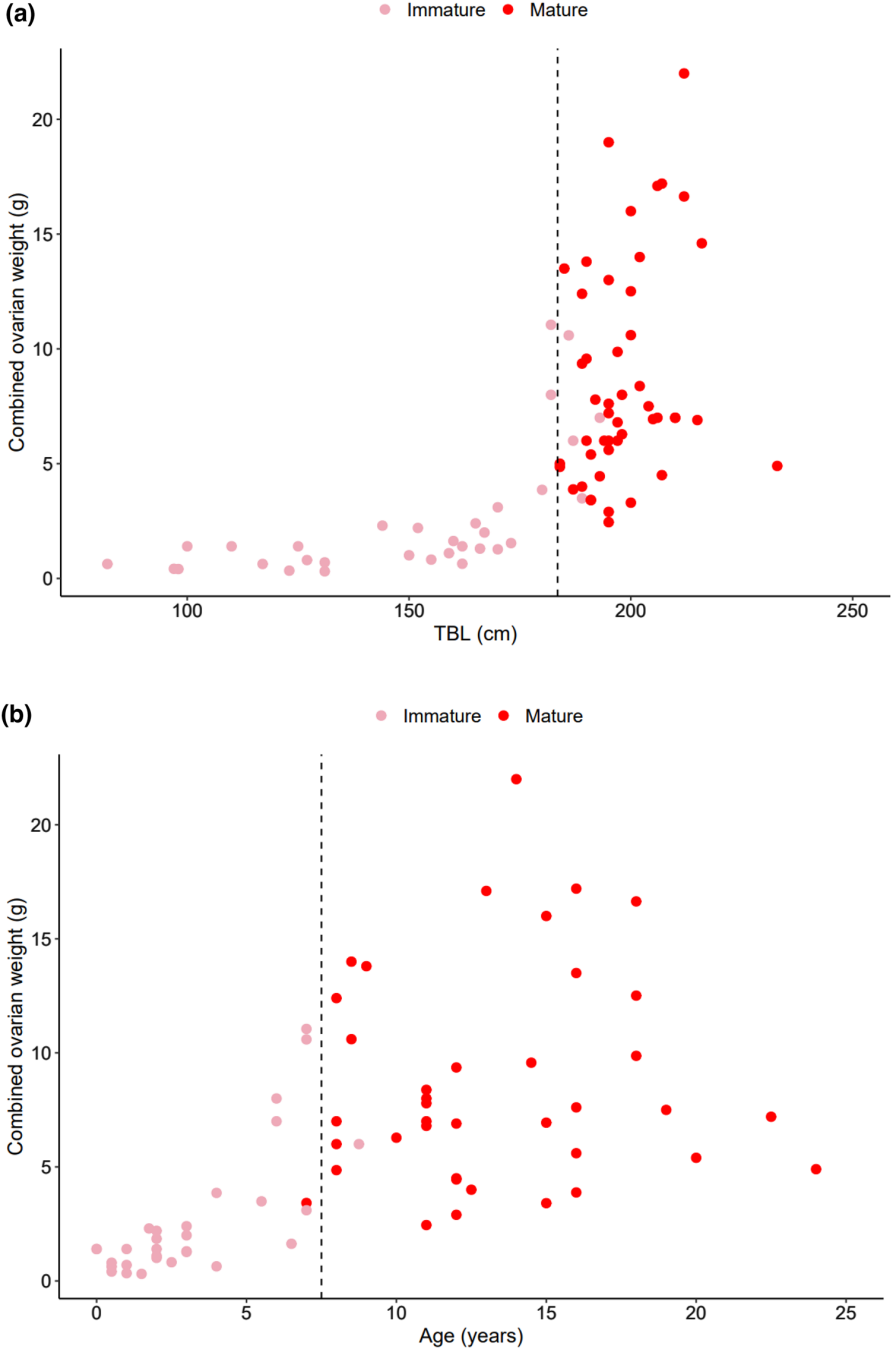
Combined ovarian weight versus **a** total body length (TBL; *n* = 104) and **b** age (*n* = 68) in female common dolphins (*Delphinus delphis*) stranded and bycaught on the New Zealand coast between 1997 and 2019. The dashed line indicates the best estimate of average length (i.e., 183.5 cm) and age (i.e., 7.5 years) at attainment of sexual maturity

Ovarian characteristics were assessed in 56 mature female common dolphins. Combined ovarian weight increased from birth until the onset of sexual maturity (7.5 yrs, 183.5 cm; Fig. 4). Combined weight of immature ovaries ranged between 0.3 and 11 g and were pale and smooth in colour and texture, respectively. Ovaries of mature females ranged from 2.5 to 22 g in weight, were ochre in colour, had visible blood vessels, and were less smooth in texture. The combined weight of mature ovaries (*x̄* = 8.7 ± 0.7 g) were significantly heavier (*t*-test, *p* < 0.001) than immature ovaries (*x̄* = 2.5 ± 0.5 g), despite overlap between the heaviest immature ovaries (11 g) and the smallest mature ovaries (2.5 g).

Combined *corpora* count recorded for sexually mature females ranged from 1 to 19 (*x̄* = 8.7 ± 0.7; *n* = 48). *Corpora* scars were observed on both ovaries in mature individuals (Table 1), though the left ovary (*x̄* = 5 ± 0.6) had significantly more *corpora* than the right ovary (*x̄* = 1.7 ± 0.4, *p* < 0.001). Of a total of 123 *corpora*, 97 were observed on the left ovary and 26 on the right ovary. The smallest *corpora* measured at 2.5 mm and the largest at 25 mm in mean diameter. The individual with the highest number of *corpora* scars (*n* = 19) also had a CL present (mean diameter of 12 mm) and was an older, resting mature, female (WS05-37Dd, 24 years). The youngest pregnant female (KS12-13Dd, 8 years) was in her first pregnancy as evidenced by a foetus present in the uterus and only one *corpora* present on the ovaries. In comparison, two other young females, KS19-23Dd (7 years) and WS06-13Dd (8.5 years), had 12 and 14 recorded CAs, respectively.

CLs were observed on the ovaries of sexually mature females in all reproductive classes. The largest were present on both pregnant, and pregnant and lactating, females (*x̄* = 27.4 ± 1.9 mm, *n* = 11, range 16–36 mm). Lactating (*x̄* = 16.3 ± 2.0 mm, *n* = 3, range 13–20 mm) and resting (*x̄* = 8.3 ± 1.9 mm, *n* = 3, range 6–7 mm) females also had CLs recorded but these were smaller in size, indicating a recent pregnancy or ovulation. As no histology was carried out on the ovaries, active CLs could not be distinguished from regressing CLs/young CAs. This may have resulted in misclassification of young CAs versus CLs. No relationship between CL size and foetal TBL was evident (*p* = 0.771, *r*^2^ = 0.01524, *n* = 8; Fig. S2).

### Persistence of corpora scars and ovulation rate

**Fig. 5:**
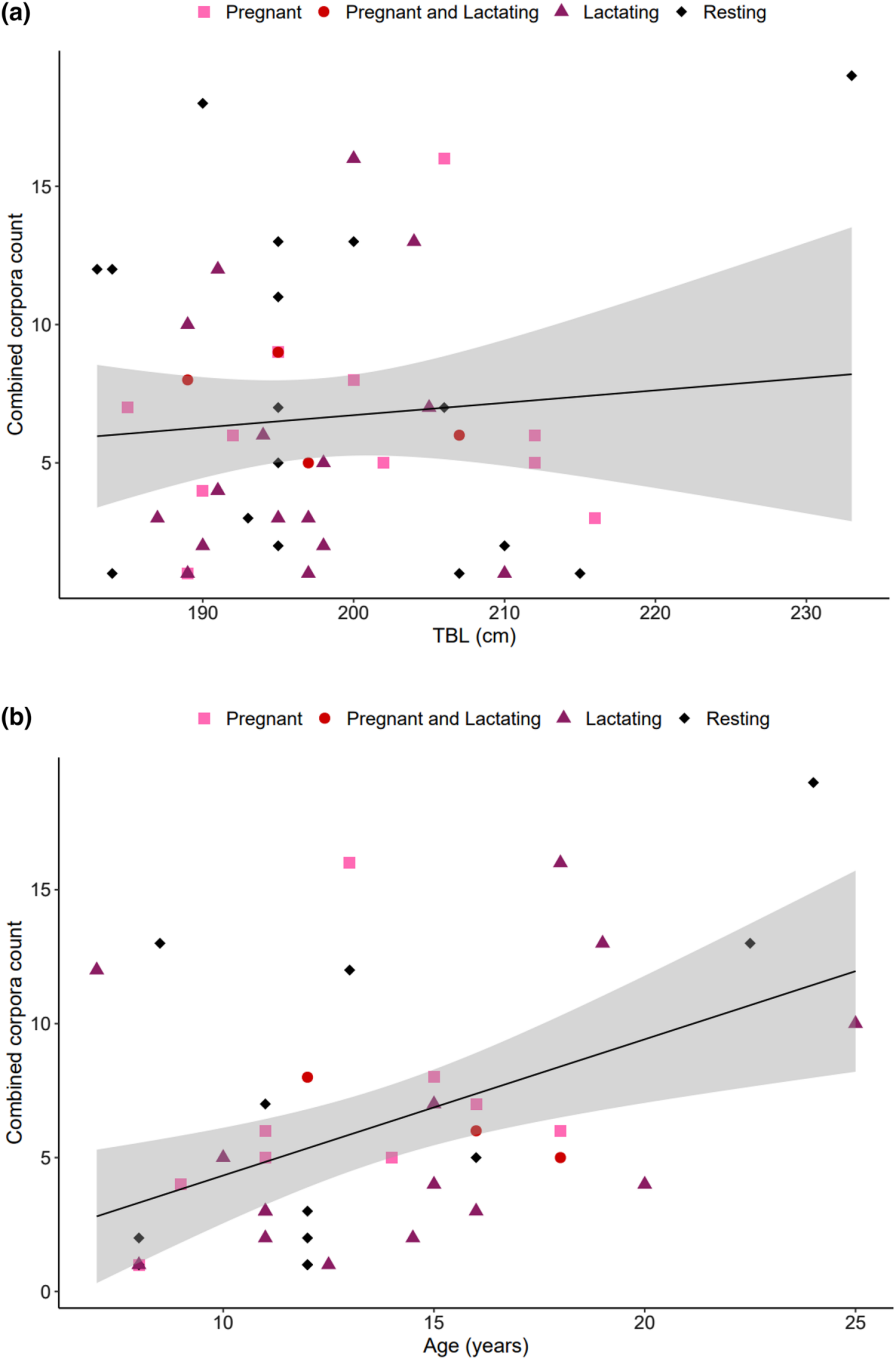
Linear regression of combined *corpora* count (ovarian activity) as a function of **a** total body length (TBL; *y* = 0.043*x*–2.109, *r*^2^ = 0.008, *n* = 49) and **b** age (*y* = 0.5085*x*–0.7539, *r*^2^ = 0.2245, *n* = 44) for mature female common dolphins (*Delphinus delphis*) stranded and by caught on the New Zealand coast between 1997 and 2019. The black solid line represents the regression, and the shaded area is the 95% confidence interval

**Fig. 6:**
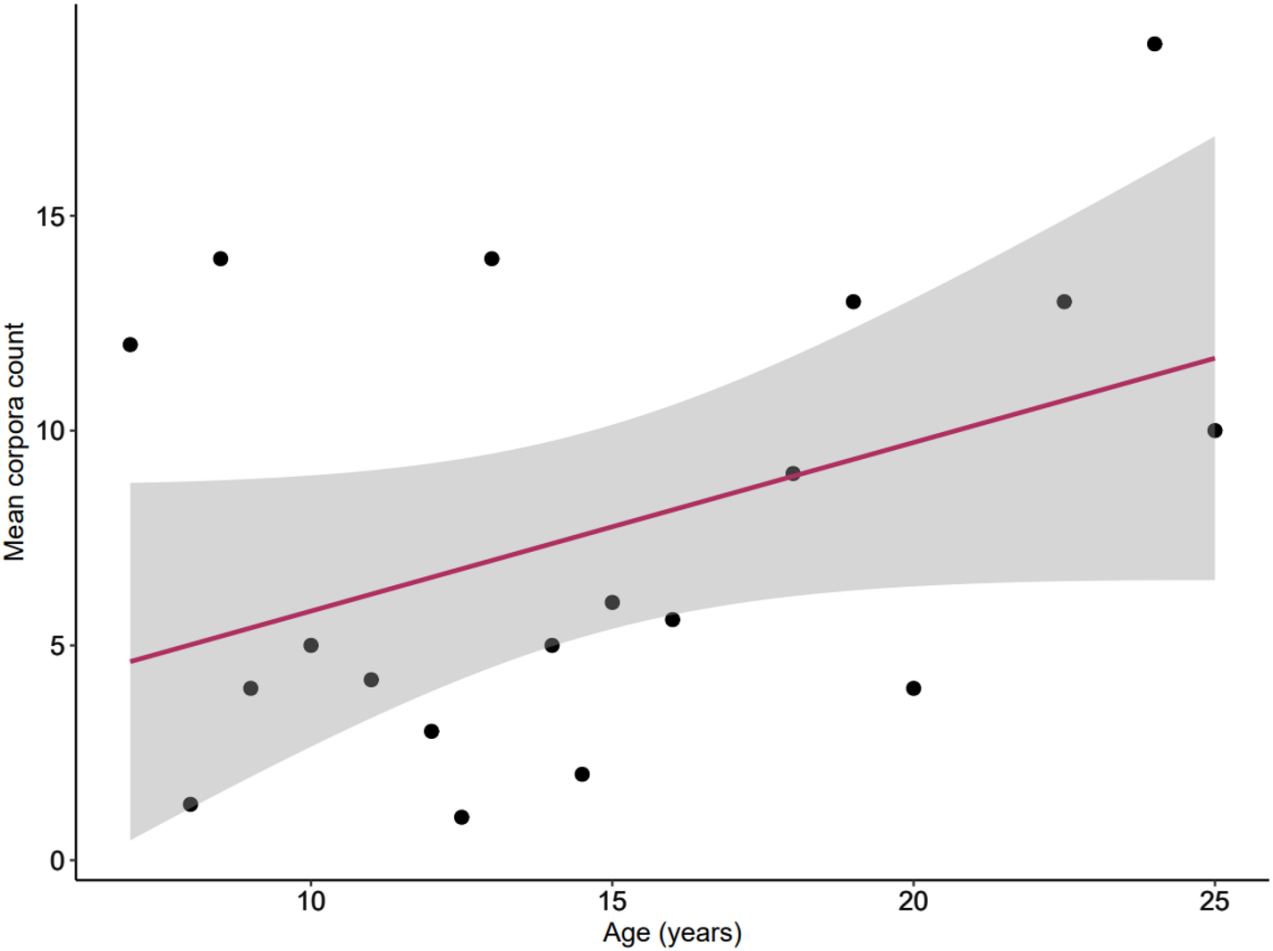
Linear regression of mean *corpora* count on age for New Zealand female common dolphins (*Delphinus delphis*) stranded and bycaught on the New Zealand coast between 1997 and 2019 (*y* = 0.3924x + 1.8754, *r*^2^ = 0.1692, *n* = 18). The solid line represents the linear regression, and the shaded area indicates the 95% confidence interval

Positive linear relationships between the number of *corpora* scars (CAs + CL) and both TBL (*r*^2^ = 0.008, *p* = 0.5412, *n* = 48, Fig. 5a) and age (*r*^2^ = 0.2245, *p* = 0.002, *n* = 39, Fig. 5b) were found, though only the relationship with age was significant. The size-frequency distribution of all CAs (*n* = 123) was plotted to further examine the persistence of *corpora* scars over time. This distribution formed a bell-shaped curve, which is indicative of a normal and complete sample (Fig. S3). A peak in the mean diameter of *corpora* at 6 mm was noted. Few, very large CAs suggest an initial period of rapid decline in *corpora* size. The ovulation rate for New Zealand common dolphins was considered to be 0.3924 *corpora* per year, i.e., the slope of the linear regression of the mean number of *corpora* scars on age (*r*^2^ = 0.1692, *p* = 0.08, *n* = 19, Fig. 6).

### Length at birth

A total of 19 foetuses were recorded in the dataset, ranging from 15.2 to 101 cm TBL (*n* = 9). The largest female foetus was 92 cm, and the largest male foetus was 101 cm. The smallest born female and male calves measured at 82 cm and 89 cm, respectively. There were 2 foetuses and 17 neonates measuring between 90 (the smallest neonate/calf) and 101 cm (the largest foetus). The median length at birth was calculated as 87.6 cm (Fig. S4, 95% CrI: 81.25–92.59 cm), based on the HOF logistic regression model.

### Gestation period, foetal growth and dates of conception and birth

**Fig. 7:**
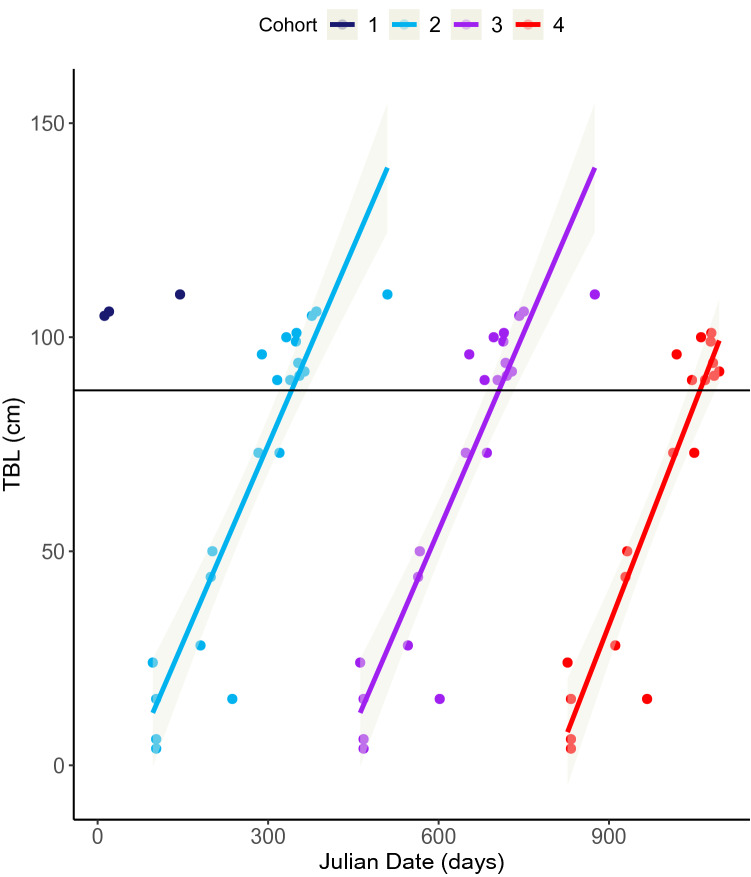
Three repetitions of assigned cohorts of a plot of foetal and neonatal total body length (TBL) against day of year sampled (Julian date) for common dolphins (*Delphinus delphis*) stranded and bycaught on the New Zealand coast between 1997 and 2019 (*n* = 22). The diagonal solid line indicates the growth trajectory for a ‘nominal’ cohort and is fitted by a linear regression. The horizontal solid line is the estimated length at birth (87.6 cm). The grey shaded areas represent the 95% CI for the linear regression

The outputs from both methods of calculating gestation period were closely aligned. The Huggett and Widdas method for estimating linear foetal growth phase of the gestation period, by regressing foetus/neonate TBL on sampling data for a nominal ‘cohort’ (*t*_g_ − *t*_0_), was estimated to be 341 days or 11.2 months (*y* = 0.3083*x*–176,531, *r*^2^ = 0.8553, *p* < 0.001, *n* = 22, Fig. 7). The length of nonlinear growth phase was calculated as 43 days (*t*_0_ = 0.126 × 341 days), which gave a total gestation period of 384 days, 12.6 months (*t*_g_ = 341 + 75 days). A foetal growth rate of 6.95 cm/month was estimated.

The regression method for calculating gestation period, which allows for low breeding synchrony/no reproductive seasonality, estimated a gestation period of 391 days or 12.8 months, based on the length at birth estimate 87.6 cm. A foetal growth rate of 6.84 cm/month was calculated for this method. For this study, both the Huggett and Widdas and the regression methods for calculating gestation period were used as although these data demonstrated some degree of seasonality, the evidence for seasonality was not conclusive.

### Lactation, length at weaning, and resting period

Length at weaning was calculated as 126.8 cm (95% CI 121.9–133.7) based on Huang et al. (2009), and an estimated female asymptotic length of 201.7 cm (95% CI 196.8–206.5; Palmer et al. unpublished data). Using fitted growth curves from examined carcasses (Palmer et al. unpublished data), the length at weaning corresponds to 8 and 9 months of age for male and female calves, respectively. This is not consistent with the lactation period which is estimated to exceed a year. Applying the Perrin and Reilly (1984) equation, the resting period was also calculated to be either 12.6 or 12.8 months, where the Huggett and Widdas and the regression methods for calculating the gestation period were applied, respectively. There were equal proportions of pregnant/pregnant and lactating females (*n* = 18), lactating females (*n* = 18), and resting mature females (*n* = 18) in our sample.

### Annual pregnancy rate and calving interval

The sample for estimating pregnancy rate was collected between 1997 and 2019 and included 56 sexually mature females, of which 18 (32%) were pregnant. The annual pregnancy rate (APR) was calculated as 30% when including all mature females. When excluding females during the peak calving period (August to November), the APR was calculated as 33% (16/48). By taking the gestation, lactation, and resting periods, 12.6 or 12.8 months each, a calving interval of either 37.8 months (3.15 years) or 38.4 months (3.2 years) was calculated. The calving interval was also calculated by taking the inverse of the APR, which gives 3.33 or 3.13 years for the two pregnancy rate estimates.

### Reproductive seasonality

Of the 27 females with a CL present, 12 were likely ovulating (based on the presence of a CL but no detectable foetus), with the majority observed over austral spring (September to November, *n* = 4) and summer (December to February, *n* = 4). However, instances in March (austral autumn, *n* = 1) and August (austral winter, *n* = 2) also occurred. Macroscopic follicles were observed in February, April, June, July, August and December (Fig. S5), with the largest follicle (> 6 mm) recorded in December.

Conception and birth dates were back-calculated for each foetus (*n* = 12) and yearling calf (*n* = 32). Using the Huggett and Widdas method for gestation, estimated conception dates were recorded in all months. There were peaks in October and November and secondary peaks in August and May (Fig. S6a). Estimated birth dates were also recorded in all months of the year with peaks in October and November (Fig. S6a). By season, austral spring (September to November) had the highest conception and birth dates (40.9% and 45.5%, respectively) and austral summer (December to February) had the lowest (both 15.9%).

Using the logistic regression method for gestation, estimated conception dates were recorded in every month of the year with the biggest peak in October and secondary peaks evident in September and July (Fig. S6b). Estimated birth dates were also recorded in all months with the biggest peak in November and secondary peaks in October and August (Fig. S6b). When observing seasons, austral spring (September to November) had the highest proportion of conception dates (40.9%) and austral summer (December to February) had the lowest (15.9%). For birth dates, austral spring had the highest (45.5%) and austral summer had the lowest (13.6%).

## Discussion

### Age and length at attainment of sexual maturity

Our LSM estimates for female common dolphins in New Zealand waters were 183.5 cm (*n* = 104) and 188.9 cm (*n* = 27) for the logistic regression and SOFI methods, respectively (S6 in Supplementary Material). These estimates both align with common dolphin populations in the eastern tropical Pacific (186.5 cm, *n* = 700; Danil and Chivers 2007) and eastern North Atlantic (*ca.* 188 cm, *n* = 453; Murphy et al. 2009), which used the SOFI and logistic regression methods, respectively. In the western North Atlantic, females attain sexual maturity at approximately 200 cm, which is over 10 cm larger than any other population (*n* = 69; Westgate and Read 2007). However, Westgate and Read (2007) do not report how the LSM for the western North Atlantic population was estimated. In the southwestern Atlantic, off the coast of Argentina, an overlap between immature and mature female TBL was noted, with the largest immature female measuring 191 cm and the smallest mature female measuring 178 cm (*n* = 35; Grandi et al. 2022). Due to a small sample size, the LSM was not estimated for the Argentine population. In contrast, the estimated LSM for female common dolphins in the North Pacific is at least 10 cm less than any other population for which estimates exist (*ca.* 172 cm, *n* = 43; Ferrero and Walker 1995).

Our estimated ASM of 7.5 years (*n* = 88) based on logistic regression for female common dolphins in New Zealand waters represents the youngest ASM reported for females in any international population (S6 in Supplementary Material). The ASM was also calculated using the SOFI method, 8.39 years (*n* = 17), and this was closer to previously reported *D. delphis* ASMs. For example, females in the North Atlantic are estimated to obtain sexual maturity at 8.22 years (regression method [Generalised Linear Model], *n* = 108; SOFI method = 8.66 years, *n* = 379; Murphy et al. 2009) and 8.3 years (SOFI method, *n* = 69; Westgate and Read 2007) for the eastern and western populations, respectively. In the North Pacific and eastern tropical Pacific, the estimated ASM is 8 years (SOFI method, *n* = 43) and 7.9 years [regression method (maximum likelihood), *n* = 405], respectively (Ferrero and Walker 1995; Danil and Chivers 2007). In the southwestern Atlantic, the oldest immature female and youngest mature female sampled were 6 years and 7 years old, respectively (*n* = 35; Grandi et al. 2022). ASM was not estimated in that study due to the small sample size. The estimate of ASM for the New Zealand population is at the lower end of what has been observed for this species (Table S7), though any differences may be due to sampling variation (and available sample size). Further research with combined datasets is required to determine the extent of any differences among populations. New Zealand samples were acquired from both stranded and bycaught individuals with an approximate 50:50 split of immature and mature females, as is consistent with other studies (e.g., Murphy et al. 2009; Westgate and Read 2007).

Age was a better indicator of sexual maturity than TBL. This has important management implications as it confirms that TBL (the easiest parameter to obtain in the field by the management agency) is not the best predictor of sexual maturity. Instead, to accurately predict sexual maturity, post-mortem collection of teeth is required from stranded or bycaught dolphins (when cultural consent from hapū is given) to enable accurate age estimation. If TBL is the only parameter able to be collected, accurate linear measurements are required. While there is a possibility that our study underestimated the age of older individuals due to the potential occlusion of the pulp cavity (Murphy et al. 2014), the standardised methods we employed, including the use of blind readings and at least two experienced readers per individual (Perrin and Myrick 1980; Murphy et al. 2014) mitigates such concerns.

### Ovarian asymmetry

In New Zealand common dolphins, 3.7 times more *corpora* scars were observed on the left ovary compared to the right. Such asymmetry has been observed in other common dolphin populations (Murphy 2004; Danil and Chivers 2007), as well as spinner dolphins (*Stenella longirostris*, Perrin et al. 1976, 1977), harbour porpoises (*Phocoena phocoena*, Murphy et al. 2010), Indo-Pacific bottlenose dolphins (*Tursiops aduncus*, Kemper et al. 2019), and long-finned pilot whales (*Globicephala melas*, Soto et al. 2017; Betty 2019). While such asymmetry exists for many species, there have been no conclusive reasons as to why this is the case in odontocetes, though ovarian asymmetry has also been observed in birds and linked to egg protection during the final stage of development (Guioli et al. 2014).

### Persistence of corpora and ovulation rate

The variability in ovulation rate as determined by *corpora* count (see Fig. 5) may suggest that some females ovulate more often than others. This has been previously observed in common dolphins (Danil and Chivers 2007) and other species including harbour porpoises (Murphy et al. 2010), long-finned pilot whales (Soto et al. 2017; Betty 2019), and Indo-Pacific bottlenose dolphins (Kemper et al. 2019). In this study, this is seen in the two young females that have unusually high *corpora* count. These females could represent a biological anomaly or may be the result of variation in ovulation rates where certain females hyper-ovulate, before they become pregnant. In common dolphins and harbour porpoises, the onset of sexual maturation can be marked by a variable number of successive infertile ovulations (Collet and Harrison 1981; Gaskin et al. 1984; Murphy et al. 2010). While such variation in ovulation exists, evidence across various odontocete species still supports the theory that *corpora* persist temporally (Perrin and Reilly 1984; Danil and Chivers 2007; Westgate and Read 2007; Betty 2019). This aligns with our findings that beyond sexual maturity, the mean number of *corpora* marginally increases. In contrast, Dabin et al. (2008) suggested most CAs would heal quickly, with a half-life of less than one year and that *corpora* may not persist over time as the number of CAs did not increase with age past age at sexual maturity in short beaked common dolphins (Dabin et al. 2008). However, recent research supports our findings as Inbaraj et al. (2021) found through an extensive literature review that most reports of *corpora* persistence in cetaceans record long-lived *corpora*. *Corpora* scars have previously been used as an index of reproductive activity over a female’s lifetime (Perrin and Donovan 1984; Murphy et al. 2010, 2018). However, future research is required to distinguish scars of ovulation from scars of pregnancy (Danil and Chivers 2007) and the rate of accumulation (Marsh et al. 1984).

### Gestation, lactation, and resting periods

We estimated gestation at 12.6–12.8 months for the New Zealand population based on the Hugget and Widdas equation and the regression equation, respectively. This is a slightly more protracted gestation period compared with international populations of *D. delphis*, which range between 10 and 12 months for the North Pacific (Ferrero and Walker 1995), North Atlantic (Westgate and Read 2007; Murphy et al. 2009), and eastern tropical Pacific (Danil and Chivers 2007). An extended gestation period intuitively signals a longer time for growth and development however, the reported length-at-birth for New Zealand common dolphins is 87.6 cm (95% CrI: 81.25–92.59 cm). Alongside eastern tropical Pacific length at birth estimates (83.7 and 87 cm), New Zealand’s is one of the smallest estimated for *D. delphis* populations, which somewhat contradicts this theory. The rate of maternal energy turnover can influence foetal brain and body growth in mammals (Barton and Capellini 2011), meaning slower growth would result in an extended gestation period. Foetal growth appears slower in New Zealand (6.84–6.95 cm/month) compared to other international (7.6 and 8.2 cm/month) *D. delphis* populations (Danil and Chivers 2007; Westgate and Read 2007; Murphy et al. 2009). Lack of reproductive seasonality and slower rates of population increase can also increase gestation length (Clauss et al. 2014, 2021). Resources must be available year-round for female common dolphins in New Zealand waters as births occur year-round, akin to what has been observed for the species in the eastern tropical Pacific (Danil and Chivers 2007). This contrasts with the North Atlantic populations where there is a distinct unimodal calving period (Westgate and Read 2007; Murphy et al. 2009). The small sample size of foetuses and neonates (*n* = 22) within the current study produces a degree of error. Additionally, small changes to the estimated length at birth may result in profound changes to the estimated length of gestation. Though it cannot be ruled out that other factors, such as exposure to persistent pollutants, may impact foetal development and therefore, result in an extended gestation period (Murphy et al. 2018).

Contaminants can pose a threat to reproduction and may affect gestation and foetal development (Murphy et al. 2015; Nabi et al. 2018; Nawrot et al. 2018). For example, a high proportion of lipophilic pollutant offload occurs from mother to offspring during pregnancy and lactation (Stockin et al. 2007; Cadieux et al. 2016; Mongillo et al. 2016). In cetaceans, transfer of organochlorides during gestation is between 3 and 15% compared to 67–99% during lactation (Mongillo et al. 2016). Transfer of contaminants from mother to calf has been documented for several odontocete species including killer whales (*Orcinus orca*, Lundin et al. 2016; Desforges et al. 2018) and beluga whales (*Delphinapterus leucas,* Béland et al. 1993; Cadieux et al. 2016). Within a New Zealand context, such maternal offloading has been recorded in bottlenose (*Tursiops truncatus*; Tezanos‐Pinto et al. 2015) and common dolphins (DDTs (dichlorodiphenyltrichloroethane) and PCBs (polychlorinated biphenyls): 42–46% lactational transfer; Stockin et al. 2007).

The lactation period of New Zealand common dolphins (12.6–12.8 months) is within the range of lactation periods estimated from previous studies; 10.35 months for the eastern North Atlantic population (Murphy et al. 2009), and 16.5 months for the eastern tropical Pacific population (Danil and Chivers 2007). Lactation periods are generally associated with body size and other life history traits (Gowans 2019). Therefore, it is common for there to be variation in reproductive parameters, such as lactation, within as well as between populations (Karniski et al. 2018).

The length at weaning estimate for the New Zealand population is 126.8 cm using the Huang et al. (2009) equation. This equation is curated with generalised linear models and based on data collected across 79 species of cetaceans including common dolphins. The length at weaning for the New Zealand population corresponds to between 8 and 9 months of age (obtained from growth models, Palmer et al. unpublished data). While less than the estimated lactation period of ~ 12.5 months reported here, this likely represents the transition period where consumption of both solids and milk occurs. Peters et al. (2020) reported a decrease in δ^15^N values (an indication of trophic position) at a body length of 160 cm for common dolphins in New Zealand, which may indicate the transition from milk to live prey. This also aligns with the estimated lactation period within our dataset, the largest 1 year old was 160 cm in length. Similarly, Chivers et al. (2016) reported calves swimming independently of their mothers at a TBL of 140.5 cm which is approximately 14 months of age (Danil and Chivers 2007). Swimming independently refers to their ability to forage and consume live prey, independent of lactation. Additional age-at-length data for females would provide a more robust estimate of weaning period as the equation is based on asymptotic length. Stomach contents for individuals around the estimated weaning age would also provide further insight.

The resting period observed for New Zealand female common dolphins ranged from 12.6 to 12.8 months, with the Hugget and Widdas and the regression methods, respectively. Considerable variation in resting periods has been noted across other studies of *D. delphis*, ranging from 2.8 months in the eastern tropical Pacific (Danil and Chivers 2007) to 20.7 months in the eastern North Atlantic (Murphy 2004; Murphy et al. 2009). Such variation could be driven by differences in habitat and resource availability, the success of previous pregnancies, and whether breeding seasonality is present in the population (Mann et al. 2000; Beauplet et al. 2006; New et al. 2013).

### Annual pregnancy rate and calving interval

The estimated APR for female New Zealand common dolphins is 30–33% which is consistent with estimates for *D. delphis* populations from the North Atlantic (Westgate and Read 2007; Murphy et al. 2009; Read et al. 2019). For example, the estimated APRs for the eastern and western North Atlantic populations are 26% and 28%, respectively (Westgate and Read 2007; Murphy et al. 2009). In the temperate eastern North Pacific, a birth rate of 13% was reported by converting the estimated proportion of calves assuming a 1:1 male:female sex ratio in the population (Chivers et al. 2016). This is much lower than a previous study in the region where the APR was recorded as 28% using steroid hormones to assess pregnancy (Kellar et al. 2006, 2014). In the eastern tropical Pacific, a higher APR of 47% has been recorded using the same methodology as used in this study (Danil and Chivers 2007).

The estimated calving interval of 3.2 years (*n* = 56) for the New Zealand population is almost half a year shorter than that recorded for the eastern North Atlantic (3.79 years, *n* = 248; Murphy et al. 2009). The shorter calving interval observed in New Zealand waters is a result of the slightly higher APR, in comparison to the eastern North Atlantic. A factor that may influence the population calving interval estimate (through impacting the pregnancy/newborn survival rate) is a female’s pollutant load. This has been suggested for the eastern North Atlantic population which is reported to have higher levels of organochlorines compared to other common dolphin populations, as well as higher rates of observed cases of reproductive pathologies and dysfunction (Murphy et al. 2018). Variation in calving intervals may also be driven by the differing environments of New Zealand waters in comparison to North Atlantic waters. Geographic variation in life history traits within a species can be driven by population-specific adaptations to local habitats (Danil and Chivers 2006; Ferguson and Higdon 2013). Differences in these parameters could also be driven by seasonal (Westgate and Read 2007; Murphy et al. 2009) or year-round reproduction (Danil and Chivers 2007).

In New Zealand, 7% (*n* = 4 out of 56) of sexually mature females were simultaneously pregnant and lactating. In the eastern North Atlantic, this was 6% (*n* = 18 out of 302; Murphy et al. 2009) and in the western North Atlantic, only one female in the dataset was pregnant and lactating (3%, out of 39 mature females; Westgate and Read 2007). In comparison, 30.4% (*n* = 65 out of 333) of mature females in the eastern tropical Pacific were both pregnant and lactating (Danil and Chivers 2007). The high proportion of simultaneously pregnant and lactating females in the eastern tropical Pacific could be explained by their elongated lactation period (16.5 months). It could also reflect a higher intrinsic reproductive rate for this population (Danil and Chivers 2007) due to the abundant resources available which enable them to maintain this energetically costly condition (Fiedler and Reilly 1994). Another explanation may be density-dependent responses exhibited by the population following a decline from decades of fisheries impacts (Gerrodette 2008; Gerrodette and Forcada 2005; Cramer et al. 2008). The New Zealand population also experiences notable levels of fisheries bycatch (Stockin and Orams 2009; Abraham et al. 2017, 2021), with 220 common dolphins reported bycaught between 2002 and 2020 (Fisheries New Zealand 2022), noting further that < 20% of the fishery was independently observed during this period. Continued monitoring will be important to identify any potential increase in the proportion of sexually mature females that are simultaneously pregnant and lactating. Populations that are at a low density tend to reproduce at a faster rate due to the availability of resources per capita (Murphy et al. 2018). This may be the case for the New Zealand population, and this idea is also supported by a comparatively lower ASM. There is also the possibility that this is the baseline/natural pregnancy rate for the New Zealand population, as no previous data exists.

The APR is typically calculated by excluding females in the mating season since it is easy to overlook early embryos and subsequently underestimate pregnancy rate. We calculated the APR with and without females during the weak calving season (October and November) and found very similar outputs. We therefore consider that the APR is reflective of the population. Future studies with an increased sample size are recommended to facilitate greater precision on reproductive seasonality and specifically, ovulation rate. In the field, pregnancy assessment of free-ranging *Delphinus* via progesterone concentration from blubber biopsies (Trego et al. 2013; Kellar et al. 2014) would also be of notable benefit to our understanding of the APR within the New Zealand population.

### Reproductive seasonality

Weak seasonality was observed in the reproduction of female common dolphins in New Zealand waters. In comparison, strong seasonality is observed, with a clear breeding season during the boreal summer months of May to September, in the Northern Hemisphere (Westgate and Read 2007; Murphy et al. 2009). Reproduction is seasonal and synchronised in these populations, which allows mothers and calves to take advantage of seasonally abundant food and resources, and warm temperatures (Rutberg 1987; Thayer et al. 2003; Westgate and Read 2007; Henderson et al. 2014). Reproductive seasonality is also observed in the North Pacific with calving peaking in boreal summer months of May and June (Ferrero and Walker 1995). Alternatively, in the eastern tropical Pacific, breeding has been documented as year-round (Danil and Chivers 2006, 2007), although further findings from the eastern North Pacific revealed calving peaks in boreal winter which corresponds with high productivity and prey biomass (Chivers et al. 2016). In New Zealand, temporal changes to primary productivity are known to affect the diet of common dolphins (Peters et al. 2020; Stockin et al. 2022). These temporal changes may also be impacting reproduction and resulting in the weak seasonality observed in the New Zealand population.

Conception and birth dates for New Zealand common dolphins were recorded in all months of the year (Fig. S6) with peaks noted between August and December (late austral winter, austral spring, and early austral summer). This indicates that while the breeding occurs year-round, there is a marginal bias with 59% of births occurring between August and December. This is supported by field evidence, where a high prevalence of neonates is observed off the eastern coast of North Island, New Zealand, specifically the Hauraki Gulf, Bay of Islands and Bay of Plenty during austral spring and summer (Neumann 2001; Schaffar-Delaney 2004; Stockin et al. 2008). The presence of calves (including neonates) year-round in the Hauraki Gulf, Auckland (Schaffar-Delaney 2004; Stockin et al. 2008), also supports the idea of weak reproductive seasonality for females in the region.

## Conclusions

Female reproductive parameters such as ASM, LSM, reproductive phases, pregnancy rates, calving intervals and reproductive seasonality play an important role in understanding the life history of a species. Here, we present data from 106 female common dolphins, comprising of 50 sexually immature and 56 sexually mature individuals. As with all opportunistic datasets, our sample size was limited and there is a possibility that this sample may not be fully representative of the population. However, our sample comprised of females from three different sources (single strandings, mass strandings, and fisheries bycatch) spanning a range of ages and maturity levels, giving us confidence in our inferences. With a baseline of reproductive parameters now established, monitoring and management can occur to allow for conservation priorities to be identified.

## Supplementary Information

Below is the link to the electronic supplementary material.

Supplementary file 1 (PDF 448 KB)

## Data Availability

The datasets generated during and/or analysed during the current study are available from the corresponding author on reasonable request.
