# Supplementary material for: Reproductive biology of female common dolphins (Delphinus delphis) in New Zealand waters
Source: Mar Biol. 2022 Nov 28;169(12):158. doi: 10.1007/s00227-022-04139-3 (PMC9705467; doi:10.1007/s00227-022-04139-3)
Supplement: Supplementary file 1 — (PDF 448 KB) [file 227_2022_4139_MOESM1_ESM.pdf]

Reproductive biology of female common dolphins (*Delphinus delphis*) in New Zealand waters

Emily I Palmer*[1], Emma L Betty[1], Sinéad Murphy[2], Matthew R Perrott[3], Adam N H Smith[4], Karen A Stockin*[1]

[1]Cetacean Ecology Research Group, School of Natural Sciences, Massey University, 0745 Auckland, New Zealand

[2]Marine and Freshwater Research Centre, Department of Natural Resources & the Environment, School of Science and Computing, Atlantic Technological University, ATU Galway City, Old Dublin Road, Galway, H91 T8NW, Ireland

[3]School of Veterinary Science, Massey University, Palmerston North, New Zealand

[4]School of Mathematical and Computational Sciences, Massey University, 0745 Auckland, New Zealand

***Corresponding authors**: e.palmer1@massey.ac.nz; k.a.stockin@massey.ac.nz

Supplementary Material

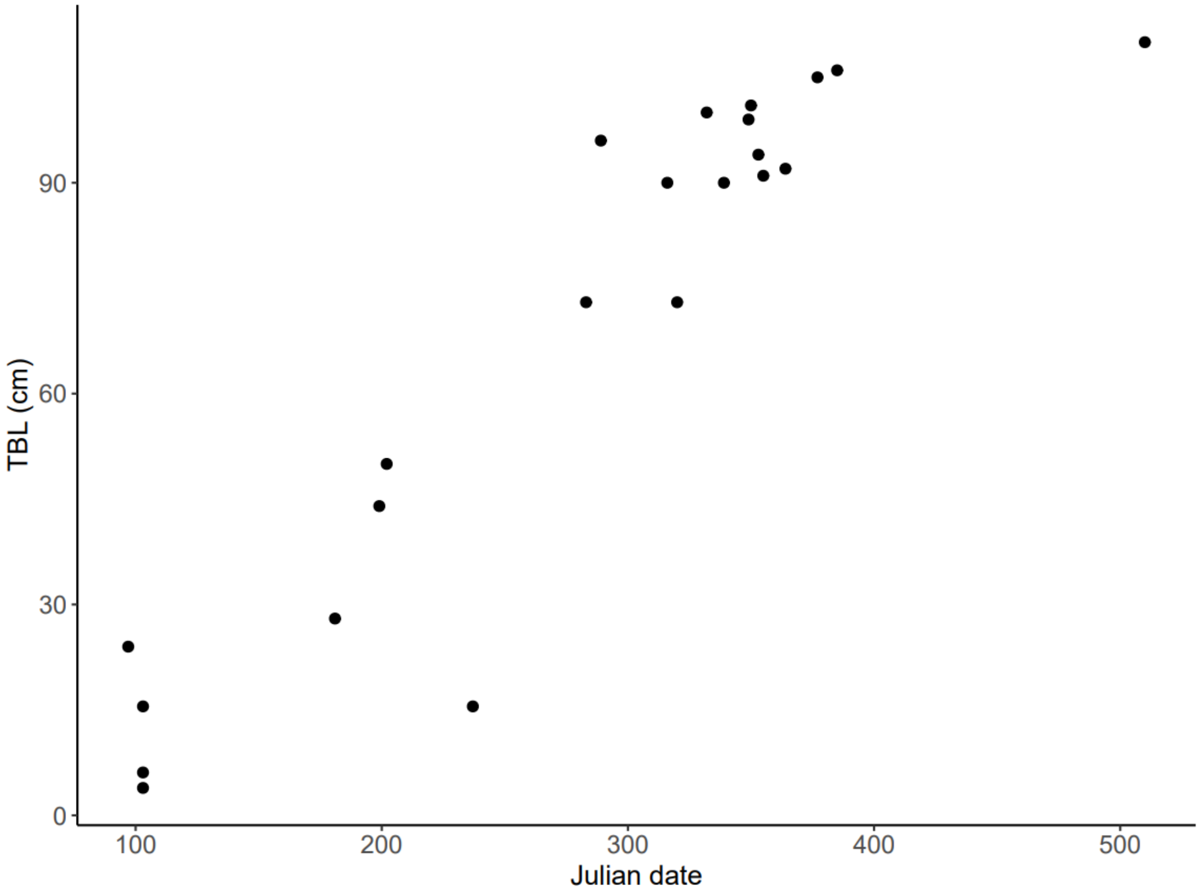

Fig. S1 Crown to rump measurement (cm) of foetuses and total body length (TBL) of neonates (cm) of foetuses and neonates against day of year of collection (Julian date) for common dolphins examined from New Zealand waters between 1997 and 2019

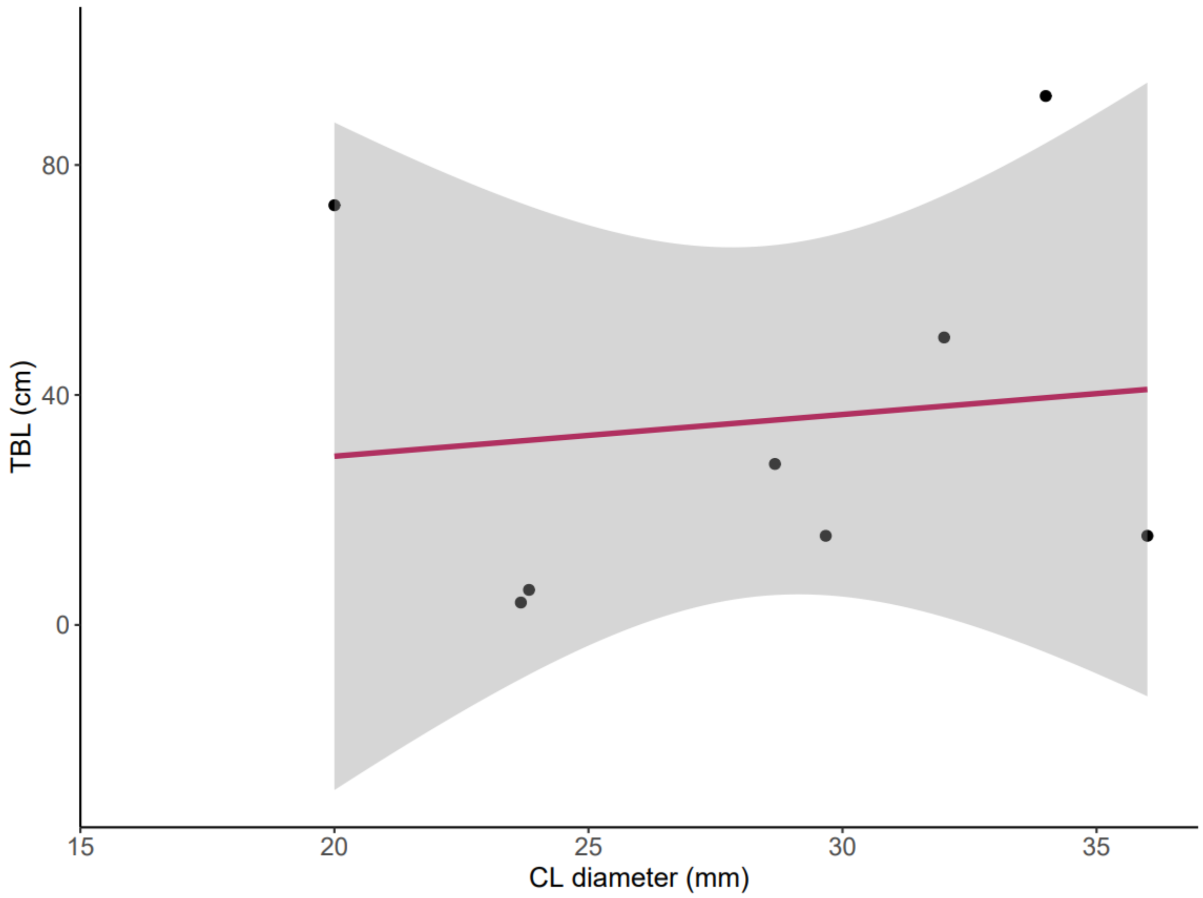

Fig. S2 Linear regression of foetal crown to rump measure (cm) as a function of *corpus luteum* (CL) diameter (mm) for female common dolphins examined from New Zealand waters between 1997 and 2019. The solid pink line indicates the linear regression (Y = 0.7263x + 14.814, r^2 = 0.01524, *n* = 8). The shaded area represents the 95% confidence interval for the regression

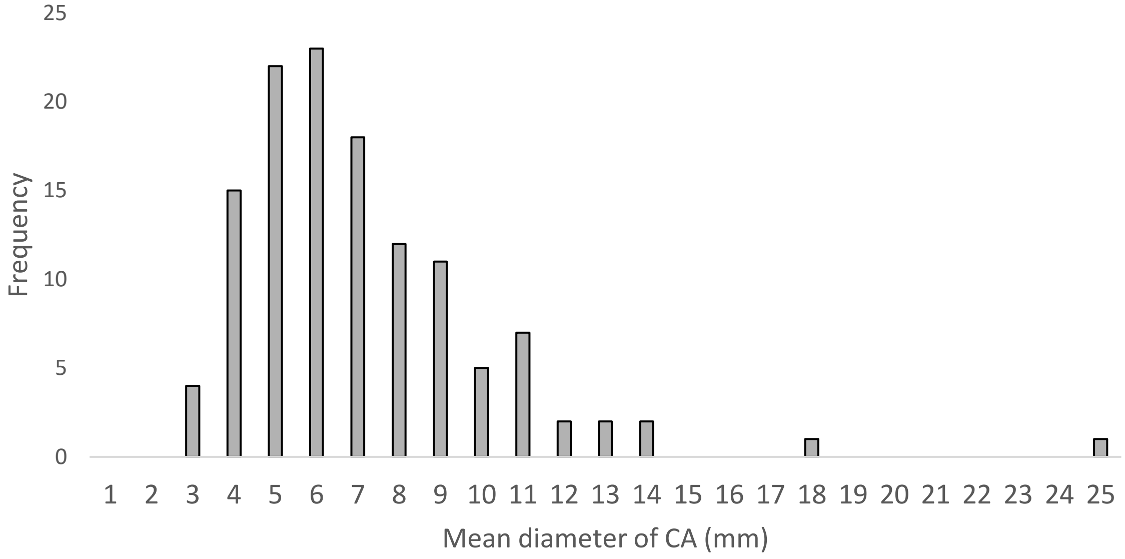

Fig. S3 Size frequency distribution of *corpora albicantia* (CAs) on the ovaries of female common dolphins (n = 123 from 49 individuals) examined from New Zealand waters between 1997 and 2019

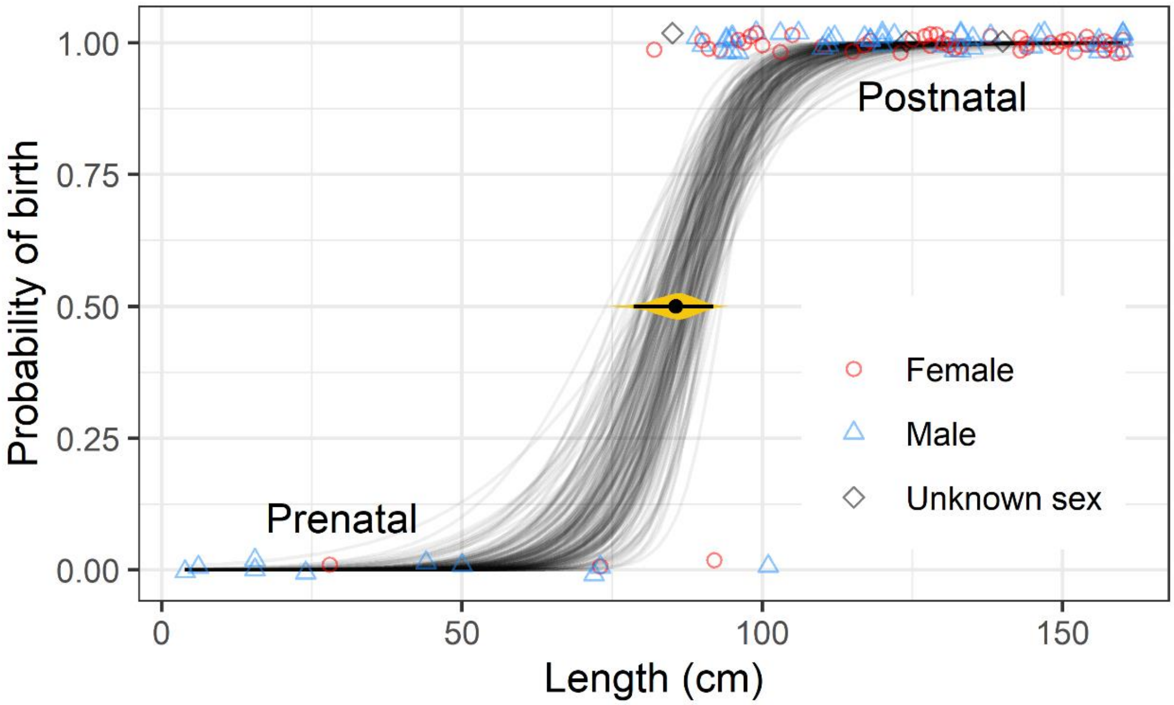

Fig. S4 The total body lengths of prenatal and postnatal common dolphins examined in New Zealand from 1997 to 2019 (*n* = 103). Logistic curves show the mean probability of birth as a function of length (thin grey lines) using the partially weighted model that disregards sex and is fitted to *n* = 103 cases. 'Jitter' and a small amount of transparency was used to aid in the visualisation of the overlapping points. The small centre point and thin horizontal line show the mean and 95% highest posterior density interval for the length at which the probability of birth is 50% i.e., the estimated median length-at-birth. The gradient plot is highlighted in yellow (Kay, 2021).

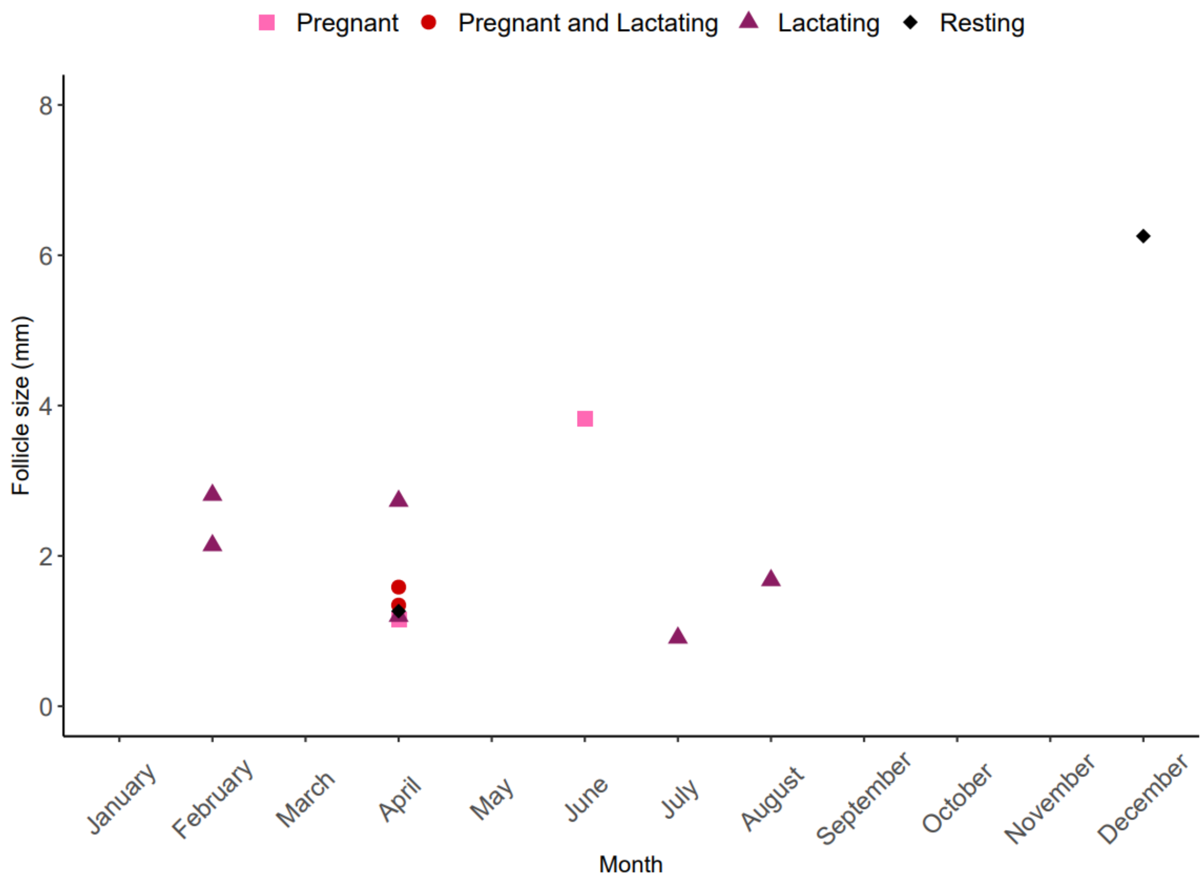

Fig. S5 Mean diameter of the largest follicle on either ovary for mature female common dolphins (pregnant, pregnant and lactating, lactating, resting mature, n = 12) examined from New Zealand waters between 1997 and 2019

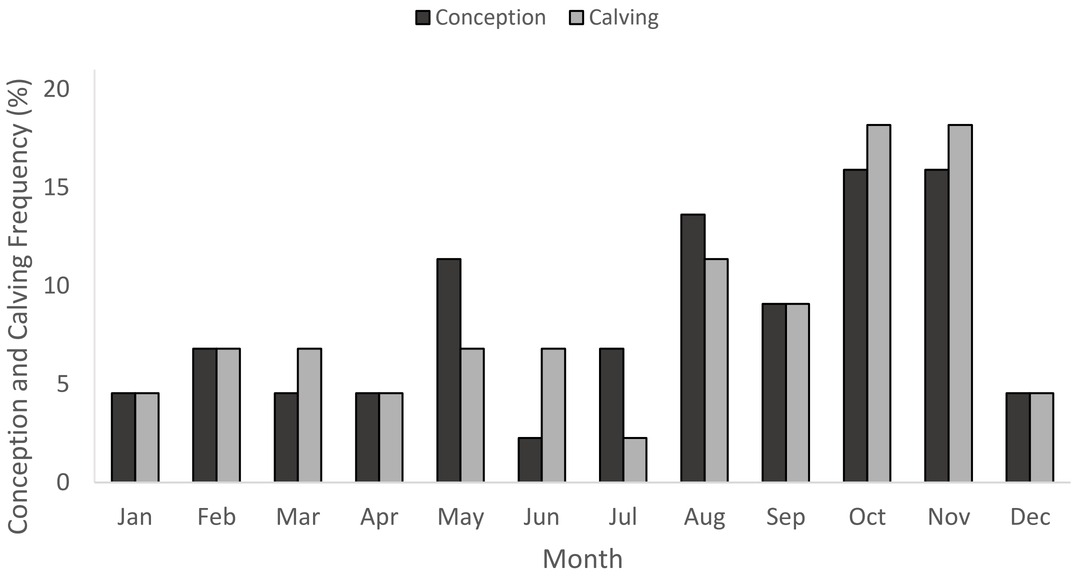

(b)

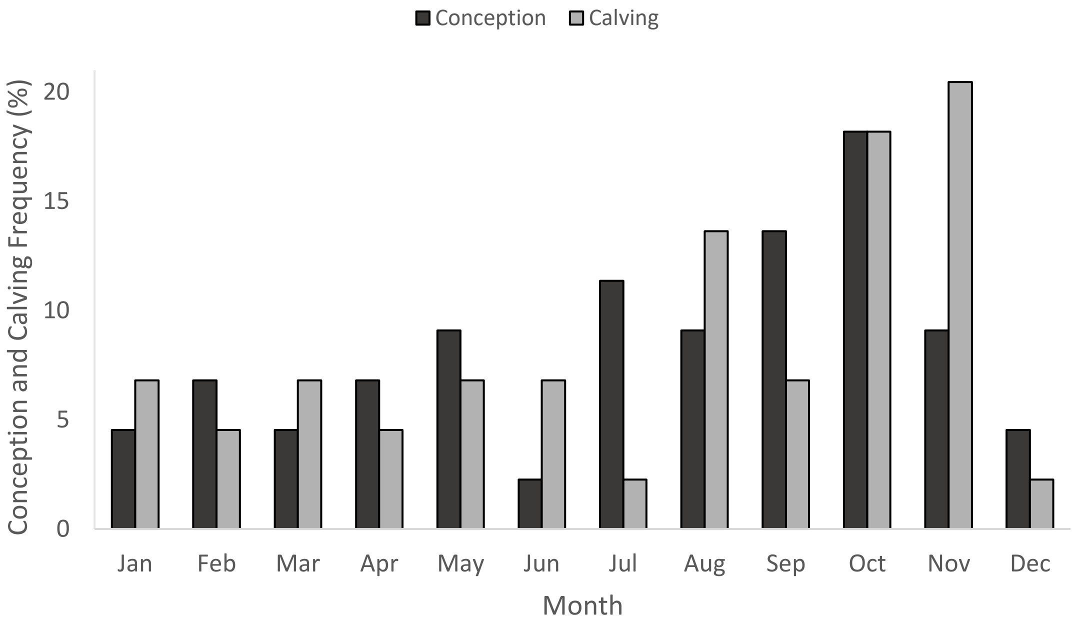

(c)

Fig. S6 Monthly distribution of estimated conception and birth dates for foetuses (n = 12) and calves <1 year of age (n = 32) using the (a) Huggett and Widdas (1951) method and the (b) regression method for calculating gestation period (Perrin et al. 1977) for common dolphins examined from New Zealand waters between 1997 and 2019

Table S7: Summary of female common dolphin (*Delphinus delphis*) estimates of average age (ASM, in years) and length (LSM, in cm) from published studies. Where available, 95% confidence (CI) or credible (CrI) intervals are also presented.

**Location**	**Eastern North Atlantic**	**Eastern North Atlantic – North West Spain**	**Western North Atlantic**	**Eastern Tropical Pacific**	**North Pacific**	**Western South Atlantic**	**New Zealand**
**Reference**	(Murphy, 2004, Murphy et al., 2009)	(Read et al., 2019)	(Westgate, 2005; Westgate & Read, 2007)	(Danil and Chivers, 2007)	(Ferrero and Walker, 1995)	(Grandi et al., 2022)	This study
**Source**	Strandings and bycatch	Strandings and bycatch	Strandings and bycatch	Bycatch	Bycatch	Strandings and bycatch	Strandings and bycatch
**ASM (95% CI or CrI)**	Regression = 8.2<br>(7.7 – 8.7)<br>SOFI = 8.7<br>(SE = 0.03)	8.4	8.3<br>(7.9 – 8.8)	Regression = 7.9<br>(7.3 – 8.3)<br>SOFI = 7.8<br>(SE = 0.02)	~8<br>Immature: 0 – 7.2<br>Mature: 8.5+	~ 7<br>Immature: 0 – 6<br>Mature: 7 – 21	Regression = 7.5<br>(6.7 – 8.3)<br>SOFI = 8.4<br>(7.3 – 9.5)
***n* ASM**	Immature = 36<br>Mature = 72	Total = 168	Immature = 32<br>Mature = 37	Total = 405	Total = 43	Immature = 10<br>Mature = 25	Immature = 42<br>Mature = 46
**LSM (95% CI or CrI)**	Regression = 188.8<br>(SE = 0.02)	187	202<br>(200.1 – 204.4)	Regression = 186.5<br>(185.5 – 187.4)<br>SOFI = 187<br>(SE = 0.01)	Regression = 170.7<br>(SE = 2.74)<br>SOFI = 172.8<br>(SE = 0.56)	Immature: 82 – 191<br>Mature: 178 – 210	Regression = 183.5<br>(179.5 – 186.5)<br>SOFI = 188.9<br>(187.9 – 189.9)
***n* LSM**	Immature = 172<br>Mature = 281	Total = 224	Immature = 32<br>Mature = 37	Immature = 349<br>Mature = 351	Total = 43	Immature = 10<br>Mature = 25	Immature = 49<br>Mature = 55
**Method used**	Regression (generalised linear model) and SOFI	Not reported	SOFI	Regression (logistic) and SOFI	Regression (logistic) and SOFI	Not reported	Regression (Bayesian logistic) and SOFI

Equation S8: sum-of-fraction immature method

The sum-of-fracture of immature method for estimating the average age at attainment of sexual maturity (ASM) was used as follows:

$$ASM = j + \sum_{i=j}^{K} p_i x_i$$

$$\text{Variance } (s^2) = \sum \frac{(p_i q_i) x_i}{N_i - 1}$$

J is the first indeterminate age class, k is the last indeterminate age class, p_i is the proportion of immature specimens in age class i, q_i is the proportion of mature specimens in age class i ($p_i + q_i = 1$), x_i is the number of age classes combined to obtain a sample size of >2 in age class i, I_i is the number of immature specimens in age class i, M_i is the number of mature specimens in age class i, and N_i is the number of specimens in age class i ($N_i = I_i + M_i$). Where, if $I_i \neq N_i$, $p_i = Ii / N_i$, and $q_i = (M_i)/N_i$; if $I_i = Ni$, $p_i = (I_i - ½)/N_i$, and $q_i = (M_i + ½)/N_i$, and if $M_i = N_i$, $p_i = (I_i + ½)/N_i$, and $q_i = (M_i - ½)/N_i$.

The average length at attainment of sexual maturity (LSM) was estimated by modifying the SOFI method, using constant length intervals (5 cm) instead of age (after Danil and Chivers, 2007).

$$LSM = j + \sum_{i=i_{min}}^{i_{max}\Sigma} p_i x_i$$

$$\text{Variance } (s^2) = \sum_{i=i_{min}}^{i_{max}\Sigma} \frac{p_i(1-p_i)x_i}{n_i-1}$$

J is the lower limit of the length class with the smallest mature animal, i_{min} is the length class with the shortest mature animal, i_{max} is the length class the longest mature animal, p_i is the proportion of immature animals in length class i, x_i is the proportion of length classes combined in length class i, n_i is the total number of animals in the ith length class.
